# Learning engages transient and sustained cellular mechanisms in the human brain

**DOI:** 10.1371/journal.pbio.3003861

**Published:** 2026-06-18

**Authors:** Guillermina Griffa, Marco Palombo, Abraham Yeffal, Hong-Hsi Lee, Agustin Solano, Susie Y. Huang, Valeria Della-Maggiore

**Affiliations:** 1 IFIBIO Houssay, School of Medicine, Department of Physiology, University of Buenos Aires, Buenos Aires, Argentina; 2 Cardiff University Brain Research Imaging Centre (CUBRIC), School of Psychology and School of Computer Science and Informatics, Cardiff University, Cardiff, United Kingdom; 3 ICIFI School of Science and Technology (ECyT), University of San Martín, Buenos Aires, Argentina; 4 Athinoula A. Martinos Center for Biomedical Imaging, Department of Radiology, Massachusetts General Hospital, Harvard Medical School, Boston, Massachusetts, United States of America; 5 Department of Neurology and Neurosurgery, McGill University, Montreal, Quebec, Canada; University of Glasgow, UNITED KINGDOM

## Abstract

Structural neuroplasticity supports learning, development, and shapes vulnerability to brain disorders, making it a central priority in neuroscience research. However, progress in humans has remained limited by the inability to probe cellular processes in vivo, leaving mechanistic insight largely dependent on animal models. To address this gap, here we combined the sub-voxel sensitivity of ultra–high-gradient diffusion MRI with the cell-compartment specificity of the Soma and Neurite Density Imaging (SANDI) model to probe structural plasticity directly in the living human brain. By tracking how learning modulates the temporal dynamics of cell bodies and cell processes, we aimed to distinguish plastic from nonplastic biological processes driving changes in microstructure. We found that learning a motor skill triggered two distinct temporal responses: a transient expansion of cell bodies across all brain regions engaged by the task, consistent with a short-lived homeostatic mechanism, and a sustained increase in cell-process density restricted to key motor regions, consistent with structural plasticity. Our approach provides a mechanistic window into human neuroplasticity and marks a significant step toward bridging the gap between animal and human neuroscience.

## Abstract 2

Understanding structural neuroplasticity in humans is challenging because in vivo methods rarely capture underlying cellular mechanisms driving learning-related brain changes. This study shows that learning induces transient cell-body expansion and sustained increases in neurite density in motor regions, revealing temporal mechanisms of human structural plasticity.

## Introduction

Experience continually remodels cellular architecture through structural modifications such as synaptogenesis, dendritic spine turnover [1,2], and the reorganization of astroglial processes that stabilize new synapses [3]. Structural plasticity supports learning and development and may influence the pathophysiology of psychiatric and neurodegenerative disorders [4–10]. Much of what is known about its cellular basis comes from invasive studies in animal models. Still, these species are evolutionarily distant and often can only approximate the complexity of human cognition and pathology. This gap underscores the need to study structural plasticity directly in the living human brain.

Diffusion MRI (dMRI) offers a promising noninvasive window into cytoarchitecture because it is sensitive to micrometer-scale restrictions imposed by the cell [11,12]. Over the past two decades, diffusion tensor imaging [DTI; 13] has been the primary approach for studying experience-dependent microstructural change in humans. In gray matter, DTI-derived mean diffusivity (MD) is sensitive to learning and correlates with synaptogenesis and astroglial expansion, two hallmarks of structural plasticity in rodents [14–17].

However, because DTI conflates signals from multiple tissue compartments, it cannot distinguish lasting remodeling from transient short-lived homeostatic responses such as swelling that alter cellular morphology without enduring structural impact [18–21]. As a result, DTI remains an indirect marker of plasticity. This lack of biological specificity limits the mechanistic interpretation of diffusion findings across learning, development, and disease.

To overcome these limitations, we leveraged the sensitivity of ultra–high-gradient dMRI [22–30] together with the specificity of the compartment-based Soma and Neurite Density Imaging (SANDI) model [31] to separate diffusion signals arising from cell bodies and cell processes. Using the motor sequence learning (MSL) task, a paradigm previously shown by our team to elicit robust functional and DTI changes [32], we tracked how these cellular compartments evolved at baseline, 30 min, and 24 h post-learning. By resolving the temporal dynamics of cell bodies and cell processes, we aimed to distinguish plastic from nonplastic biological mechanisms driving changes in microstructure. SANDI revealed two distinct cellular responses: a transient increase in apparent cell-body density, consistent with a short-lived homeostatic response, and a sustained rise in apparent cell-process density, compatible with structural remodeling. Our approach moves the field beyond descriptive microstructural changes toward mechanistic inference, with broad implications for development and disease.

## Results

**Fig 1:**
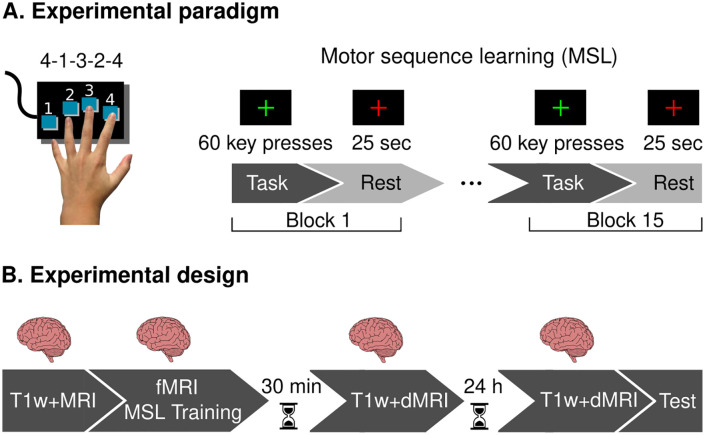
Experimental paradigm and design. **A)** Experimental paradigm. Right-handed participants performed a motor sequence learning (MSL) task involving a 5-item sequence of finger movements on a keyboard, using the four fingers of their non-dominant hand (4-1-3-2-4, with 4 representing the index finger and 1 representing the pinky finger). Initially, subjects memorized the sequence and were then instructed to execute it as quickly and accurately as possible during task blocks (green cross), and relax during rest periods (red cross). A total of 15 blocks were completed. **B)** Experimental design. fMRI images were obtained during the MSL task following a block design. To assess structural plasticity induced by learning, ultra–high multi-shell dMRI and corresponding T1-weighted images (T1w) were acquired longitudinally at three time points: before learning, 30 min post-learning, and 24 h post-learning. Finally, to assess overnight offline gains, participants completed 8 additional practice blocks 24 h after training; the test was assessed after the MRI session.

The MSL paradigm used here consists of executing a five-element finger sequence, initially learned declaratively, with the nondominant hand [Fig 1A, 33]. In our previous study, this task produced reliable improvements in performance alongside well-characterized functional MRI (fMRI) activation patterns and a distinct temporal pattern of reduction in MD in the hippocampus and precuneus [32]. To investigate the cellular mechanisms underlying these microstructural changes, here we replicated the same paradigm and longitudinal design inside the Connectome scanner [300 mT/m; 25,29,34,35], acquiring ultra–high-gradient multi-shell dMRI at baseline, 30 min, and 24 h later (Fig 1B).

**Fig 2:**
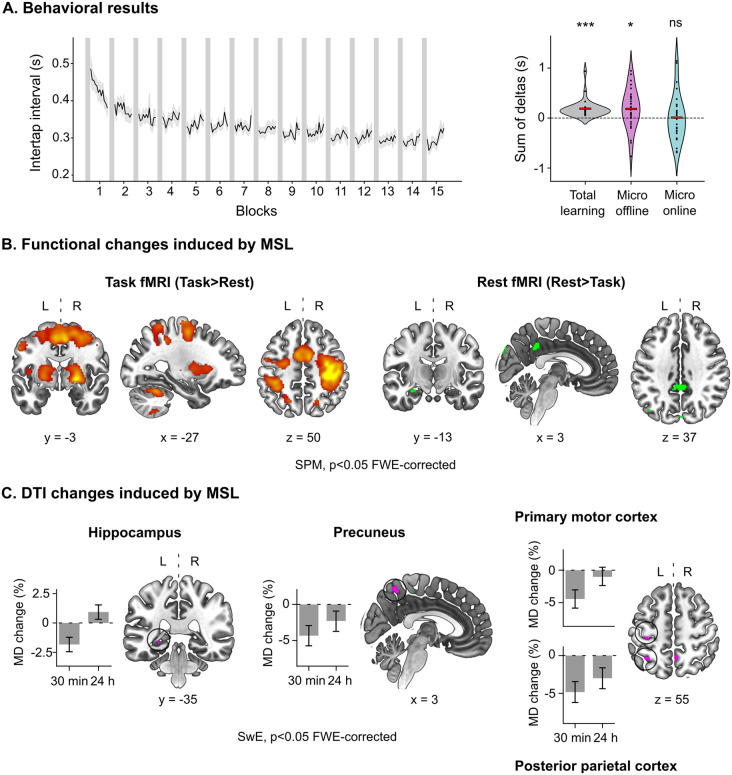
Behavioral, functional, and DTI changes induced by motor sequence learning (MSL). **A)** Behavioral results - The left panel shows the MSL curve, depicting the intertap interval -the time elapsed between successive key presses from correctly executed sequences- as a function of blocks (~12 sequences per block). The right panel shows the metrics corresponding to micro-offline gains (MOGs), micro-online gains (MONGs), and total learning, which were obtained based on the cumulative sum of deltas in performance during practice and rest blocks. Specifically, MOGs represent the difference in the mean intertap interval between the last correct sequence of a block and the first correct sequence of the next block. In contrast, MONGs represent the difference between the first and last correct sequence within a block. Total learning = MONGs + MOGs. Note that, in line with our previous work, improvements in performance on the MSL task occurred during rest periods interleaved with practice (*t* test against zero; *p* = 0.034), rather than during sequence execution (*p* = 0.59). Nonsignificant (ns), ***p < 0.001, *p < 0.05 corrected by Bonferroni. **B)** Functional results - Shown are the whole-brain voxelwise statistical parametric maps (SPMs) for the Task (Task > Rest; left plot) and Rest (Rest > Task; right plot) conditions (*p* < 0.05, Family-Wise Error [FWE]-corrected). Note that cortico–cerebellar and cortico–striatal networks were more active during task execution (in red), whereas the hippocampus and the precuneus were more active during the rest periods (in green). **C)** DTI results - Shown are the results from the Sandwich Estimator (SwE) statistical model on mean diffusivity (MD) using a whole-brain threshold-free cluster enhancement (TFCE) approach (*p* < 0.05 FWE-corrected) across the three dMRI sessions (baseline, 30 min, 24 h); barplots represent the mean % change of MD relative to the baseline ± 95% confidence intervals (CI) for the clusters identified in the whole-brain analysis. Note that MD changes are expressed as % of the baseline to facilitate the comparison to our DTI analysis carried out in a clinical MRI scanner [32]. MSL-induced a rapid (30 min) reduction in MD over the left posterior hippocampus, right precuneus, left primary motor cortex (M1), and left posterior parietal cortex (PCC). Remarkably, while hippocampal and M1 changes were transient, returning to baseline within 24 h, those in the precuneus and PPC persisted overnight. The data underlying this figure can be found at https://zenodo.org/records/20527091.

Before applying SANDI, we first examined if the dataset obtained in the Connectome scanner reproduced the behavioral, fMRI, and DTI results of our previous work conducted on a clinical system. MSL showed the expected performance improvements, dominated by micro-offline gains (MOGs) [36,37] during rest periods between practice blocks rather than during active execution (Fig 2A). fMRI analysis revealed increased activity in the left hippocampus and precuneus during rest periods, consistent with early engagement of the limbic system, while cortico–striatal and cortico–cerebellar circuits supported sequence execution throughout training (Fig 2B). Finally, MD also decreased 30 min after learning in the left hippocampus and right precuneus, with additional reductions in left primary motor cortex (M1) and posterior parietal cortex (PPC), two brain regions central to motor skill acquisition [38,39]. Critically, MD changes in hippocampal and M1 were transient, whereas those in precuneus and PPC persisted overnight (Fig 2C).

In sum, these results replicate our previous behavioral, fMRI, and DTI findings obtained from a study comparing a learning group to a nonlearning control group performing repeated, non-sequential finger tapping [32]. This replication provides support for the learning specificity of the microstructural changes reported here and strengthens the basis for their cellular-level interpretation.

### SANDI uncovers distinct cellular contributions to MD dynamics

**Fig 3:**
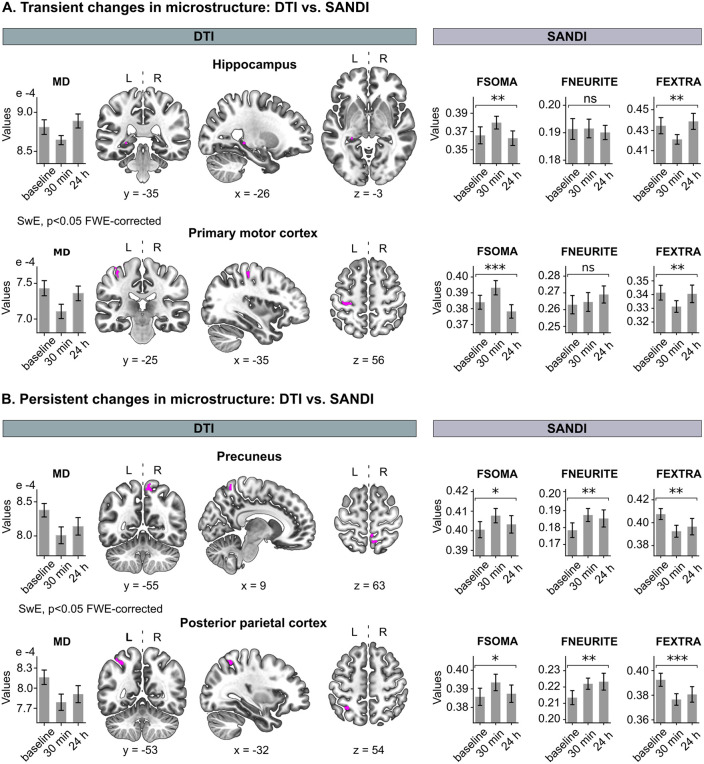
Temporal dynamics of microstructural changes induced by MSL: DTI vs. SANDI. **A)** The MSL task induced a transient decrease in MD (left panel) in the left posterior hippocampus and left primary motor cortex (SwE, *p* < 0.05 FWE-corrected). These changes were accompanied by a transient increase in apparent cell-body density (FSOMA; right panel; repeated-measures ANOVA, Bonferroni-corrected; hippocampus: *p* = 0.016; M1: *p* < 0.001). **B)** MSL also induced a long lasting reduction in MD (left panel) in the precuneus and posterior parietal cortex (SwE, *p* < 0.05 FWE-corrected), which was associated with both a transient increase in FSOMA (right panel; precuneus: *p* = 0.044; PPC: *p* = 0.043) and a sustained increase in apparent cell-process density (FNEURITE; right panel; repeated-measures ANOVA, Bonferroni-corrected; precuneus: *p* = 0.024; PPC: *p* = 0.0071) that persisted at least 24 h post-learning. Bar plots depict the time course of the mean MD/SANDI metrics ± 95% CI for each brain region. *p < 0.05, and **p < 0.05; ***p < 0.001 Bonferroni-corrected. The data underlying this figure can be found at https://zenodo.org/records/20527091.

The time course of MD changes across brain regions (Fig 2C) may reflect different phases of the same plasticity cascade or distinct underlying biological mechanisms. Because MD results from the aggregate contribution of multiple tissue compartments, DTI alone cannot distinguish between these hypotheses. To resolve this ambiguity, we applied the SANDI model to the multi-shell dMRI data and examined its compartment-specific estimates within the DTI-defined clusters (Fig 3A and 3B). SANDI is a compartment-based model that decomposes the diffusion signal in gray matter into three fractions: cell soma (FSOMA), cell processes (FNEURITE), and extracellular liquid (FEXTRA), enabling a biologically grounded interpretation of how microstructural changes evolve as a function of time. These compartment-specific measures are further supported by converging evidence from histological atlases, biophysical simulations, and preclinical studies, suggesting sensitivity to cell-body density and underlying microstructural organization [40–47].

We found that the MD reductions observed 30 min after learning were accompanied by a transient increase in FSOMA across all four regions (left hippocampus, right precuneus, left M1, left PPC; Fig 3A and 3B). Given the short time window, such changes are incompatible with processes like neurogenesis [48,49] and instead point to a rapid, reversible enlargement of cell bodies. The anatomical uniformity of this response across regions with different cytoarchitectonic and embryonic origins is consistent with a non-specific homeostatic mechanism. We propose that learning-induced neural activity causes membrane depolarization, transiently altering the ionic balance of the interstitial space. This perturbation is expected to trigger compensatory responses to buffer the ionic imbalance through cellular uptake, with water following osmotically into the cells and producing transient cell swelling [18–21]. Supporting this interpretation, all four regions were functionally engaged during learning (see Fig A in S1 Text), indicating that FSOMA changes occurred within an active network. The return of FSOMA to baseline at 24 h is consistent with the expected restoration of ionic balance toward physiological levels.

In contrast, sustained MD reductions in the precuneus and PPC were associated with an increase in FNEURITE 30 min post-learning that persisted overnight, consistent with an increment in cell-process density (Fig 3B). This pattern aligns with structural plasticity observed in animal models, specifically, with the remodeling of dendritic and astroglial processes leading to new synapses and their stabilization, respectively [2,3,7,10,50,51]. Notably, FEXTRA closely tracked MD changes across all four regions (Fig 3A and 3B), consistent with the interpretation proposed by Assaf and colleagues that MD reductions partly reflect reduced interstitial space due to cellular expansion during plasticity-related states [15,17,52].

To examine whether these compartment-specific changes related to learning, we conducted linear mixed-effects models assessing the relationship between FSOMA and FNEURITE and behavioral measures of learning (see Methods). We found a significant association between the time course of FNEURITE and MOGs (F(1,25) = 6.15, *p* = 0.020). Specifically, there was a positive relationship at both 30 min and 24 h (β 30 min = 0.007, β 24 h = 0.02), which was significantly stronger at 24 h than at 30 min (time point × MOG interaction: F(1,79) = 3.95, *p* = 0.05). This suggests that participants showing greater early consolidation underwent more persistent structural remodeling of cell processes. No significant association was found between FNEURITE and overnight memory retention, and no significant interactions between transient FSOMA changes and any behavioral metric were observed, consistent with its interpretation as a nonlearning-specific, activity-dependent response.

Together, these findings are consistent with the hypothesis that learning triggers at least two distinct biological mechanisms not distinguishable with DTI alone: a transient homeostatic response affecting cell bodies, and a sustained, regionally specific remodeling of cell processes compatible with structural plasticity. The ability to make this distinction rests on the spatiotemporal dissociation between FSOMA and FNEURITE, whose divergent temporal profiles and contrasting spatial distributions provide the inferential basis for interpreting these compartments as reflecting fundamentally different biological processes. The proposed mechanisms likely represent only a subset of the microstructural changes engaged during early learning, as processes occurring at finer spatial or temporal scales may not be detectable with the SANDI model under the current acquisition parameters.

### Independent, cross-model validation of SANDI

In the whole-brain analyses, SANDI was applied within DTI-defined clusters, maximizing sensitivity but precluding an independent test of cross-model correspondence. To address the potential circularity of this approach, we performed a complementary model-agnostic analysis in the hippocampus. This region was selected because its subfields (head, body, tail) can be reliably segmented longitudinally [53], providing an anatomically defined framework independent of diffusion metrics. DTI and SANDI were fitted independently in each subfield at each time point (baseline, 30 min, 24 h) and their topographical distributions (MD versus FSOMA/FNEURITE) and temporal dynamics were compared.

**Fig 4:**
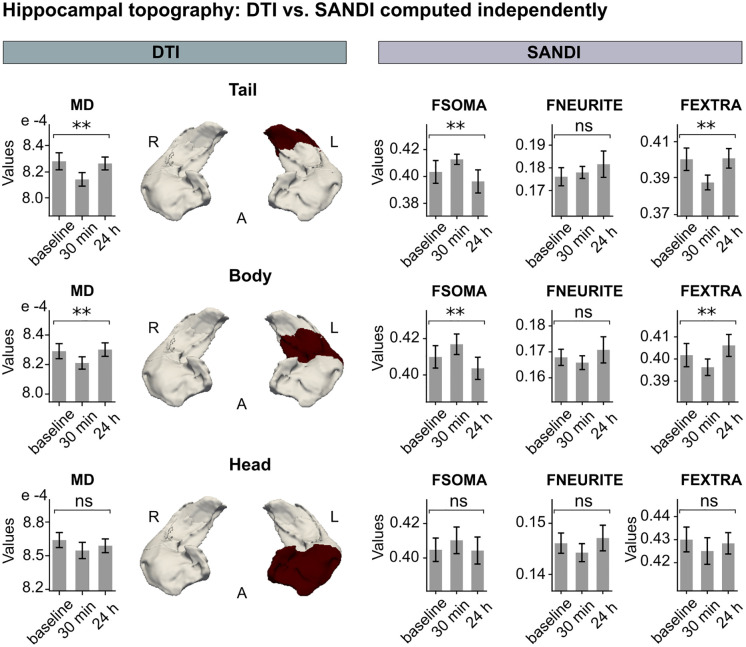
MSL-induced microstructural changes assessed independently using DTI and SANDI. Both MD and SANDI metrics were computed separately across the tail, body, and head subfields of the hippocampi (highlighted in red), which were segmented based on each subject’s T1-weighted images [53]. We observed anatomical overlap for MD and FSOMA metrics across the body and tail of the left hippocampus (repeated-measures ANOVA, Bonferroni-corrected; tail: MD: *p* = 0.0034; FSOMA: *p* = 0.022; body: MD: *p* = 0.034; FSOMA: *p* = 0.026). Notably, no changes were identified by either model in the hippocampal head or in the right hippocampus. MSL did not affect apparent cell-process density in any of the subfields (*p* > 0.05). Bar plots depict the time course of mean MD/SANDI metrics ± 95% confidence intervals (CIs). **p < 0.05; ns = nonsignificant. The data underlying this figure can be found at https://zenodo.org/records/20527091.

We observed a strong anatomical correspondence between MD and FSOMA across the three hippocampal subfields (Fig 4). Specifically, transient decreases in MD in the tail and body of the left hippocampus, previously reported in MSL and other learning tasks [32,54], were matched by a transient increase in cell-body density. Notably, no significant changes were detected in either the head or the right hippocampus, and MSL did not modulate cell-process density in any subfield. These findings are consistent with the whole-brain spatiotemporal pattern identified by DTI over the left posterior hippocampus.

The shared topography between MD and FSOMA confirms cross-model convergence and supports the robustness of SANDI for detecting structural changes in gray matter.

## Discussion

Structural plasticity is instrumental to learning, development, and vulnerability to neurological and psychiatric disorders [55–60]. However, despite decades of research, our mechanistic understanding in humans has remained limited by the inability to probe cellular processes noninvasively. By combining ultra–high-gradient diffusion MRI with the compartment-based SANDI model [31], here we show that the temporal evolution of cell compartments provides relevant information regarding the biological mechanisms supporting learning, linking microstructural dynamics to human brain function. Importantly, converging evidence from histological atlases and preclinical models supports the interpretation of SANDI-derived metrics as reflecting underlying cellular microstructure [40–47].

Prior diffusion MRI work has relied on indirect indices such as MD, which are sensitive to learning but cannot distinguish plastic changes from non-plastic phenomena such as swelling [14–17,32,52,54,61,62]. Multi-compartment models were introduced to improve biological specificity [23,27,63–67], but most rely on white-matter–based geometries and are not optimal for studying gray matter plasticity. By focusing on cellular compartments SANDI provides great potential to explore plasticity in gray matter.

Our results show that the mechanisms underlying changes in MD are likely to be heterogeneous and compartment-specific. A key feature of our approach is the longitudinal design, which enabled us to track the temporal dynamics of two cellular compartments, soma and neurites, across three timepoints spanning the early and later phases post-learning. It was precisely the combination of divergent temporal profiles and contrasting spatial distributions—the uniform FSOMA response across cytoarchitectonically distinct regions versus the regionally restricted FNEURITE increase—that provides the inferential basis for distinguishing two biologically distinct processes. We hypothesize that the widespread transient increase in cell-body density reflects homeostatic swelling of neurons and astrocytes that may be triggered by increased activity during early phases of synaptic potentiation [15,18–21]. In contrast, the persistent increase in cell-process density observed in precuneus and parietal cortex is consistent with remodeling of dendritic and glial cell processes leading to new synaptic connections and synaptic stabilization [2,50,51,68,69]. This spatiotemporal dissociation represents a significant conceptual shift in human neuroscience from descriptive diffusion changes toward identifying their biological drivers

Persistent increases in cell-process density were restricted to the precuneus and PPC, while the hippocampus and M1 showed only transient FSOMA changes. This temporal dynamics—whereby the hippocampus supports early encoding and reactivation [32,70] while structural remodeling emerges selectively in cortical regions implicated in sequence integration and long-term representation [71,72,73]—is consistent with the systems consolidation framework [74,75], whereby memories initially dependent on the hippocampus are gradually consolidated in distributed cortical networks. Notably, cell-process density increases in the precuneus and PPC were already detectable at 30 min post-learning, indicating that cortical structural remodeling is initiated rapidly after learning. This challenges the classical view of systems consolidation as a slow, sleep-dependent process and is consistent with previous work showing that hippocampal-cortical interactions begin immediately after learning [32,76]. While sleep may further stabilize the structural remodeling initiated during waking, our design and acquisition parameters do not allow us to directly assess its contribution, nor to determine whether our findings are compatible with the synaptic homeostasis hypothesis [SHY, 77], as FNEURITE is unlikely to be sensitive to the fine synaptic changes SHY predicts [31,78].

The ability to dissociate homeostatic from plastic cellular responses is not only relevant for understanding learning, but opens broader possibilities for investigating structural plasticity across development, neurodegeneration, and neuropsychiatric disorders. For example, differentiating transient from persistent cellular changes could help identify biomarkers that distinguish adaptive remodeling from pathological processes such as neuroinflammation or atrophy. Combined with emerging MRI methods that should improve sensitivity to swelling and cell-type discrimination [79–83], this approach may contribute to a biologically interpretable framework for understanding how experience, maturation, and degeneration remodel the brain’s cellular architecture.

Despite these advances, several limitations should be considered when interpreting the present findings. SANDI-derived metrics do not provide direct quantification of cellular compartments, but rather model-based inferences about their relative contributions to the diffusion signal. As such, they cannot be directly extrapolated to histological measures of cellular density or volume. In particular, due to the model’s assumption of negligible water exchange between compartments and the relatively long diffusion times used for dMRI acquisition (23 ms), neurite density values are systematically lower than histological estimates—a recognized limitation of diffusion models at these timescales [78]. As a consequence, the neurite compartment is primarily sensitive to larger cellular processes such as axons and dendrites, and is unlikely to capture fine synaptic structures, thereby defining the biological scale at which our inferences apply. Critically, because all analyses were performed relative to each region’s pre-learning baseline, this limitation does not affect the interpretation of the within-subject changes that form the basis of our conclusions. Another consideration is that SANDI yields relative signal fractions rather than absolute proton densities, making the compartment estimates compositionally dependent (i.e., the three fractions are constrained to sum to one within each voxel). More quantitative mapping would require diffusion-relaxometry acquisitions with longer scan times [84,85], calibrated to a proton-density reference such as CSF [86]. Finally, while the present findings provide evidence for distinct mechanisms driving MSL, they are based on a specific experimental paradigm in a healthy young adult sample, and may not generalize to other tasks or cohorts. Manipulating key training parameters -such as duration, intensity, or the implicit/explicit nature of the task- is likely to modulate the magnitude and persistence of compartment-specific changes, as suggested by recent work from our group [54].

In conclusion, tracking the spatiotemporal evolution of cellular compartments provides a biologically grounded framework to interpret microstructural changes in the human brain. By enabling the dissociation of plastic and homeostatic processes, this approach overcomes key limitations of traditional diffusion models and moves the field beyond descriptive changes toward mechanistic inference, thereby helping bridge cellular mechanisms identified in animals with human brain function.

## Methods

### Participants

Twenty-nine healthy human subjects between 18 and 36 years old (16 female, mean age ± SD = 26.86 ± 5.41 years) were recruited under approval by the Institutional Review Board of Mass General Brigham (IRB protocol number: 2018P002443). All procedures were conducted in accordance with the principles expressed in the Declaration of Helsinki. Written informed consent was obtained from all participants. All participants reported no psychiatric, neurological, or cognitive impairment, nor any history of sleep disturbances. All subjects were right-handed as assessed by the Edinburgh Handedness Inventory [87].

### Experimental paradigm

Both the learning paradigm and the experimental design used in this study were identical to our previous MSL study [32]. Participants were instructed to learn a 5-item motor sequence using the four fingers of their nondominant (left) hand (4-1-3-2-4, 4 being the index finger and 1 being the pinky finger) as quickly and accurately as possible (Fig 1A).

### Experimental design and procedure

All participants performed the MSL task in a self-paced manner during 15 blocks of 12 sequences each (60 key presses) interleaved with rest periods of 25 s [32]. The task lasted around 15–20 min. Multi-shell Diffusion MRI, T1-weighted, and functional images were acquired throughout the study. fMRI was collected during the MSL task following a block design (task, rest), while dMRI and T1w images were obtained in a longitudinal design at three time points: before practice (baseline), 30 min, and 24 h after learning to track the dynamics of structural plasticity in the short- and long-term (Fig 1B). Finally, to assess overnight offline gains, participants completed 8 additional practice blocks 24 h after training; the test was assessed after the MRI session.

### MRI acquisition

Diffusion MRI, T1-weighted, and fMRI images were acquired on the original 3T Connectome MRI scanner (MAGNETOM CONNECTOM, Siemens Healthcare, Forchheim, Germany), equipped with a maximum gradient strength of 300 mT/m and a slew rate of 200 T/m/s, using a 64-channel phased array head coil [88].

Functional images (BOLD) were obtained following a block design during MSL (15 blocks of self-paced practice alternated with 25 s rest). The acquisition parameters were chosen to match our previous MSL study [32]: voxel size = 3 × 3 × 3 mm^3^; FOV = 210 mm; 42 slices aligned with the AC-PC line; 10% gap; posterior-anterior (P-A) phase-encoding direction; repetition time (TR) = 1,440 ms; echo time (TE) = 30 ms; multiband acceleration factor = 2; no PAT; bandwidth (BW) = 1,786 Hz/Px; echo spacing = 0.62 ms; EPI factor = 70; flip angle = 69°. A gradient echo (GRE) fieldmap was also acquired for correction of field inhomogeneities using the following parameters: 42 slices; 10% gap; voxel size = 3 × 3 × 3 mm^3^; field-of-view (FOV) = 210 mm; posterior-anterior (P-A) phase-encoding direction; TR = 716 ms; TE1 = 4.92 ms; TE2 = 7.38 ms; flip angle = 55°; BW = 600 Hz/px.

T1w and dMRI images were acquired at baseline, 30 min, and 24 h after training. T1w images were obtained using the multi-echo sequence and the following parameters: TR = 2,530 ms; TE = 1.15 ms; flip angle = 7°; Inversion Time (TI) = 1,100 ms; BW = 649 Hz/Px; FOV = 210 mm; voxel size = 1 × 1 × 1 mm^3^; parallel acquisition = GRAPPA mode, acceleration factor = 3. The acquisition was performed in sagittal slices. The T1w images were used for the coregistration and subsequent normalization of fMRI images and for the segmentation of each subject’s hippocampal subfields.

Multi-shell dMRI images were acquired using a monopolar pulsed-gradient spin-echo, single-shot echo-planar-imaging sequence [89] using the following parameters: diffusion time = 24 ms; gradient pulse duration = 8 ms; TR = 4,200 ms; TE = 55 ms; voxel size = 2 × 2 × 2 mm^3^; partial Fourier factor = 6/8, GRAPPA acceleration factor = 2; multiband acceleration factor (MB) = 2; anterior-posterior (A-P) phase-encoding direction. dMRI images were acquired in 8 b-values: 32 gradient directions for b-values = 200, 500, 1,200, 2,400 s/mm^2^, 64 directions for b-values = 4,000, 6,000, 8,000 s/mm^2^, and 22 interspersed nondiffusion-weighted b = 0 images. The latter were acquired for signal normalization and motion correction, of which five were acquired with reversed phase-encoding direction (posterior-to-anterior) for susceptibility distortion correction.

### MRI preprocessing

#### Functional MRI.

Preprocessing of functional images was conducted primarily using Statistical Parametric Mapping (SPM12, Wellcome Department of Cognitive Neurology). Initial corrections addressed gradient nonlinearity [90]. The functional time series was motion-corrected using the middle volume of the series as a reference. Since the experimental paradigm consisted of a block design, we did not perform slice-timing correction. Susceptibility distortion due to magnetic field inhomogeneities was corrected using the phase and magnitude images of a gradient-echo field map, and the realignment was performed to the middle volume of each functional time series with the aid of FSL’s (FMRIB Software Library) “fslsplit” and “fslmerge” commands. T1w structural images were coregistered to the BOLD images and normalized to the standard MNI152 template. This transformation was subsequently applied to the BOLD data, and the functional images were smoothed using a Gaussian kernel with an 8-mm full-width at half-maximum.

#### T1.

Human hippocampal subfields were segmented using FreeSurfer (version 7.0). Each subject’s T1w image was processed through FreeSurfer’s ‘recon-all’ pipeline. The hippocampal head, body, and tail were then extracted along the longitudinal axis using the optimized ‘segmentHA_T1’ function [53].

#### Diffusion MRI.

Preprocessing and normalization of the diffusion MRI images were performed using the FSL (University of Oxford) (version 6.0.3), MRtrix3 (version 3.0.3), and Advanced Normalization Tools (ANTs; Wellcome Department, UCL) (version 2.4.2).

Preprocessing steps were applied separately for each scanning session (baseline, 30 min, and 24 h post-training). Images were denoised using MRtrix3’s “dwidenoise”, corrected for Gibbs ringing using MRtrix3’s “mrdegibbs”, and corrected for the nonlinearity of gradients [90]. Geometric susceptibility distortion correction was made using FSL’s “Topup”. Subsequently, head motion, eddy currents, and b-vector rotation were made using FSL’s “eddy” [91]. Finally, bias correction was then applied using MRtrix3’s “dwibiascorrect”.

Following preprocessing, FSL’s “dtifit” was used to fit the diffusion tensor model, generating scalar maps for fractional anisotropy (FA) and MD metrics. FA and MD maps were calculated for each subject and each voxel using the 1,200 s/mm² b-shell. Additionally, the SANDI model was fitted to the multi-shell dMRI acquisition using the SANDI MATLAB toolbox (https://github.com/palombom/SANDI-Matlab-Toolbox) [31]. SANDI accounts for cellular contributions to gray matter microstructure by modeling cell bodies as spheres, neurites as sticks, and extracellular space as isotropic Gaussian diffusion, producing three additive fractions. FSOMA tackles the contribution of the cell-body to the diffusion signal; FNEURITE estimates the contribution of cell processes such as neurites and cell processes from glial cells; FEXTRA, which represents the extracellular fluid, is calculated based on the other two fractions (FSOMA + FNEURITE + FEXTRA = 1).

To quantify the DTI and SANDI metrics for each hippocampal subfield in native space (Region of Interest –ROI analysis), MD maps and SANDI-derived fractions were registered to each subject’s T1w image using a linear registration algorithm from ANTs. On the other hand, for the whole-brain analysis, DTI and SANDI maps were normalized to stereotaxic space (MNI152) using a custom-made pipeline based on ANTs to minimize across-session test-retest reproducibility error [92]. Finally, the normalized DTI and SANDI maps were smoothed using FSL’s smoothing function with a 4-mm full-width at half-maximum Gaussian kernel.

### Data analysis and statistics

#### Behavior.

MSL was quantified using the intertap interval, defined as the time elapsed between successive key presses during correctly executed sequences [32]. The mean intertap interval was calculated for each correct sequence (~12 sequences per block) and each subject. Then, the intertap intervals from correct sequences were averaged across all subjects for each block.

Gains in performance during MSL were assessed as in our previous study [32] into micro-online gains (MONGs), micro-offline gains (MOGs), and total learning. MONGs were calculated as the difference (delta) between the mean intertap interval of the first and last correct sequence within a practice block, while MOGs were computed as the difference between the mean intertap interval of the last correct sequence of a practice block and the first correct sequence of the following block. Total learning represented the sum of MONGs and MOGs.

To statistically assess gains in performance for each of these behavioral metrics, we performed *t* tests against zero, with p-values corrected for multiple comparisons using Bonferroni adjustments.

#### Functional MRI.

Statistical analyses of BOLD images were conducted using SPM12. We performed a standard whole-brain voxelwise general linear model (GLM) analysis to identify brain regions more active during motor execution (Task > Rest) and during rest periods (Rest > Task). To determine whether the parameter estimates (beta weights) from this GLM were significantly different from zero, we applied a one-tailed t tes*t* for each condition (*p* < 0.05 corrected for family-wise error–FWE). One subject was excluded from the functional analyses due to a technical inconvenience during acquisition affecting most of the dataset.

Additionally, to assess whether the regions showing DTI/SANDI changes were functionally engaged during learning, we extracted the mean BOLD signal during early learning (blocks 1–7) from the four ROIs identified by the DTI analysis and compared it to a reference baseline period. This reference corresponded to the last 30 s of a 1-min fixation interval at the end of the scanning session, during which participants remained still viewing a neutral fixation cross. The first 30 s were excluded to ensure that any residual motor activity following task completion had dissipated. Although brief, this interval provides a reasonable approximation of a resting-state baseline. For each participant, the mean BOLD signal during Task and Rest blocks was normalized by subtracting the fMRI baseline value, and one-tailed one-sample *t* tests against zero (*p* < 0.05) were used to determine whether each region showed significant activation above baseline during Task, Rest, or both conditions.

#### Diffusion MRI.

To assess overall changes in MD across sessions (baseline, 30 min, and 24 h after learning), we conducted a whole-brain longitudinal analysis using a nonparametric permutation-based F test, using the threshold-free cluster enhancement (TFCE) approach (H = 2, E = 0.15) [93] implemented with the Sandwich Estimator (SwE) toolbox (version 2.0.0). SwE was specifically designed for accurate modeling of longitudinal and repeated-measures neuroimaging data [94]. The SwE analysis was conducted based on 1,000 permutations, and significant clusters were identified using an FWE-corrected *p*-value < 0.05. To reveal the temporal dynamics for each cluster of the F test, we extracted the median MD for each cluster, subject, and session, expressed it as the percent change relative to the baseline (Fig 2C) and determined the 95% confidence intervals using the summarySEwithin function from the Rmisc package in R [95,96]. Note that % MD changes are shown in Fig 2C to facilitate the comparison between our DTI analysis carried out in a clinical MRI scanner [32] and that acquired at the Connectome I scanner.

To distinguish between short- and long-term changes in microstructure, we conducted two additional whole-brain analyses on the same MD maps using the same SwE approach described above. We then extracted the SANDI metrics (FEXTRA, FNEURITE, and FSOMA fractions) from the MD clusters, which were used as ROIs to assess longitudinal metrics of these changes (repeated-measures ANOVA; *p* < 0.05, corrected by Bonferroni). To reveal the temporal dynamics for SANDI metrics, we then computed each fraction’s median for each cluster, subject, and session and the corresponding 95% CI (Fig 3A and 3B). Additionally, we conducted linear mixed-effects models (LMMs) to examine the relationship between the time course of FSOMA and FNEURITE changes (relative to baseline: 30 min – baseline and 24 h – baseline) and behavioral measures of learning (MOGs; micro-online gains, MONGs; and overnight offline gains). Separate models were run for FSOMA and FNEURITE, with time point (30 min, 24 h) as a repeated factor, behavioral metrics as fixed effects, and subjects modeled as a random effect, accounting for between-subject variability. For FSOMA, the key term of interest was the time point × behavioral metric interaction, consistent with a transient response. For FNEURITE, the key term was the main effect of the behavioral metric, consistent with a more persistent structural change.

To contrast DTI and SANDI across hippocampal subfields (Fig 4), we carried out independent statistical analyses (repeated-measures ANOVA; *p* < 0.05, corrected by Bonferroni) for each metric in the tail, body, and head hippocampi used as ROIs [53]. One subject was excluded from the statistical analysis due to missing data at the 24 h post-training MRI session.

## Supporting information

S1 Text Functional activation during early learning. (DOCX)

## Abbreviations

ANTs: advanced normalization tools
DTI: diffusion tensor imaging
FA: fractional anisotropy
FOV: field-of-view
FSL: FMRIB Software Library
FWE: family-wise error
GLM: general linear model
MD: mean diffusivity
MOGs: micro-offline gains
MONGs: micro-online gains
MSL: motor sequence learning
PPC: posterior parietal cortex
SANDI: soma and neurite density imaging
SPMs: statistical parametric maps
TFCE: threshold-free cluster enhancement.
