# Supplementary material for: Learning engages transient and sustained cellular mechanisms in the human brain
Source: PLoS Biol. 2026 Jun 18;24(6):e3003861. doi: 10.1371/journal.pbio.3003861 (PMC13298990; doi:10.1371/journal.pbio.3003861)
Supplement: S1 Text — (DOCX) [file pbio.3003861.s001.docx]

**Functional activation during early learning**

To assess whether the regions showing SANDI changes were functionally engaged during learning, we extracted the mean BOLD signal across blocks 1–7 (early learning) for the four ROIs identified by the DTI analysis: L hippocampus, L posterior parietal cortex, precuneus and L primary motor cortex (Fig 3A and 3B) and compared it to a reference period.

As depicted in Fig A, all four regions showed activity above this reference period in both conditions (one-tailed t-test against zero; hippocampus Rest: p < 0.001, Task: p = 0.004; M1 Rest: p = 0.003, Task: p < 0.001; PPC Rest: p = 0.004, Task: p < 0.001; precuneus Rest: p = 0.002, Task: p = 0.001), suggesting that these regions were engaged during both Task and Rest periods, albeit to different extent. Importantly, this overlap between dMRI and fMRI findings suggests that both changes in FSOMA and FNEURITE took place in brain regions that were metabolically active during learning. The transient and spatially widespread FSOMA increase is therefore consistent with activity-dependent processes linked to energy demand, such as ionic buffering (leading to swelling). In contrast, the more sustained and spatially restricted increase in FNEURITE likely reflects structural remodeling processes that evolve over a longer timescale. We cannot speculate about the relationship between FNEURITE and the BOLD signal beyond its anatomical overlap.

**
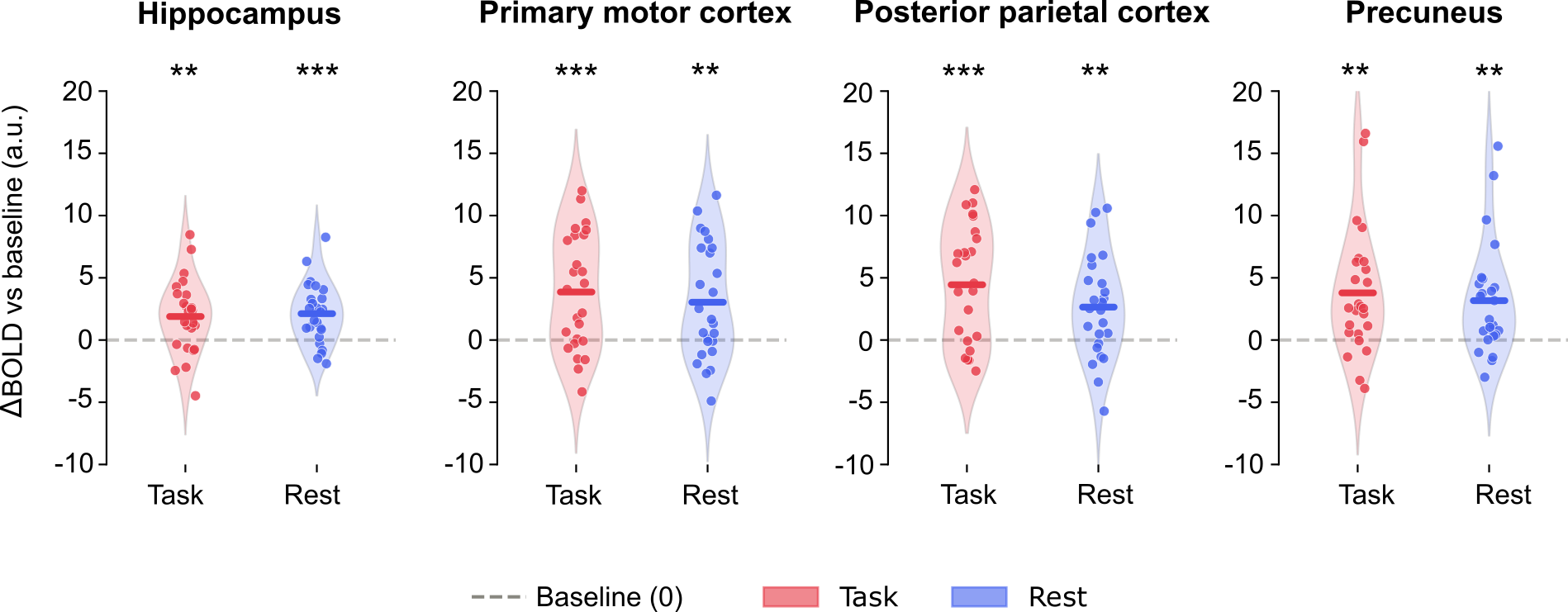
**

**Fig A - Functional activation during early learning. Violin plots show the mean change in BOLD signal relative to a resting-state baseline for the four ROIs identified by the DTI analysis (hippocampus, M1, PPC, and precuneus) during Task (red) and Rest (blue) conditions across the first 7 blocks of practice (early learning). Dots represent individual participants; horizontal lines indicate the group mean; dashed lines indicate the baseline. Asterisks indicate significant activation above baseline (one-sample t-test against zero; **p < 0.01, ***p < 0.001).**
