# Supplementary material for: Learning engages transient and sustained cellular mechanisms in the human brain
Source: PLoS Biol. 2026 Jun 18;24(6):e3003861. doi: 10.1371/journal.pbio.3003861 (PMC13298990; doi:10.1371/journal.pbio.3003861)
Supplement: Attachment — Submitted filename: Authors_response.pdf [file pbio.3003861.s004.pdf]

We would like to thank the reviewers for their thoughtful and constructive feedback on our manuscript, *"A mechanistic window onto human neuroplasticity through cell-specific diffusion MRI".* We appreciate their insightful comments and the overall positive tone of the reviews. Addressing these points has helped us refine the conceptual framing of the study, sharpen the interpretation of our findings, and better delineate the scope and limitations of our inferences, enhancing the overall quality of our work.

Below, we provide a detailed, point-by-point response addressing each of their concerns, along with a description of the corresponding revisions made to the manuscript.

Reviewer #1 (Yaniv Assaf): This is a strong and conceptually important manuscript that makes a clear advance in human neuroplasticity research. By combining ultra-high-gradient diffusion MRI with the SANDI model, the authors provide one of the first in vivo demonstrations that learning-related microstructural changes in the human brain can be temporally and compartmentally dissociated into transient, non-plastic processes (cell-body expansion) and sustained, region-specific structural plasticity (increased cell-process density). This directly addresses a long-standing limitation of diffusion MRI studies, which have been unable to distinguish swelling or homeostatic responses from genuine remodeling.

Methodologically, the work is rigorous and well controlled. The longitudinal design, replication of prior behavioral/fMRI/DTI effects, use of ultra-high-gradient data, and convergent validation via hippocampal subfield analyses substantially strengthen confidence in the findings. The interpretation is biologically grounded, well integrated with animal literature, and avoids overstatement. Importantly, the results move the field beyond descriptive diffusion changes toward mechanistic inference, which is particularly valuable for studies of learning, development, and disease.

The main limitations (model assumptions, relative—not absolute—compartment fractions, and reliance on a single learning paradigm) are appropriately acknowledged and do not undermine the central conclusions. Any remaining issues are largely matters of framing and clarification, particularly with respect to the biological specificity of the model and the extent to which these findings can be generalized beyond the present task and acquisition regime. These do not represent conceptual or technical weaknesses of the study.

Overall, this is a high-quality and high-impact contribution that is very well aligned with the scope and standards of PLOS Biology. I therefore strongly recommend acceptance, with at most minor revisions focused on sharpening the discussion and boundaries of interpretation, rather than on additional analyses or changes to the core conclusions.

We thank the reviewer for this thoughtful and positive assessment of our work and for appreciating the conceptual advance of the study.

Following the reviewer's suggestion, we have refined the Discussion to delineate more clearly the biological interpretation and limits of the SANDI-derived measures. In

particular, we explicitly state that SANDI metrics provide model-based estimates rather than direct quantification of cellular densities, and therefore cannot be directly extrapolated to histological measures, and we clarify the biological scale at which these inferences apply (Discussion, sixth paragraph).

We have also added a paragraph addressing the generalizability of our findings, noting that the present results are based on a single learning session within a specific motor learning paradigm in a healthy young adult cohort, and that extending these findings to other tasks, training regimens, and populations remains an important direction for future work (Discussion, sixth paragraph).

Reviewer #2: This is an interesting and fun manuscript to read. It makes a valuable contribution by showing that different aspects of an MRI (diffusion) signal can provide insight into different cellular processes, which are differentially affected by learning. It is a nice mix of technical development (validation) along with more cognitive/biological issues. Many interesting questions arise from these insights that I don't articulate here because they are beyond the scope of the current work; nonetheless, they speak to the importance (and potential impact) of the work.

We appreciate the reviewer's positive and constructive feedback.

There are several aspects that I struggled to understand, and so the work could benefit from clarification. These center around two major issues: (a) the relationship between the recorded signal and cellular changes; (b) new insights provided into motor memory processing. The following questions sometimes deal with one or other of these questions or at times are a bit overlapping.

1. Perhaps the greatest support for the two signals (FSOMA and FNEURITE) tapping into different biological processes is that they dissociate across time (and space). The FNEURITE signal as would be expected last longer (present at 24 hours) and for specific brain areas. It should be possible to highlight this important strength more in the work. Whilst the logic that these signals are tapping into different biological processes is sound there is not much evidence in the study that they are --- other than this very nice temporal dissociation.

We agree with the reviewer that the temporal dissociation between FSOMA and FNEURITE is the cornerstone of our interpretation. We would like to emphasize that both the temporal and spatial dimensions of this dissociation are equally informative. It was precisely the combination of divergent temporal profiles and contrasting spatial distributions — the uniform FSOMA response across cytoarchitectonically distinct regions versus the regionally restricted FNEURITE increase — that provides the inferential basis for distinguishing two biologically distinct processes. Following the reviewer's suggestion, we have emphasized this point throughout the Results and Discussion sections (Results: second and sixth paragraphs of the section "SANDI uncovers distinct cellular contributions to MD dynamics"; Discussion: third paragraph).

2. What is the relationship between the functional activation (see Fig 2 - having these was a nice strength to the work) and the FNEURITE and FSOMA signals? Are they overlapping, and so come about due to energetic changes, and therefore consistent with the (energetic) cellular changes that are thought to be the basis for the different (MRI) signal changes?

We thank the reviewer for this insightful question. To assess whether the regions showing SANDI changes were functionally engaged during learning, we extracted the mean BOLD signal across blocks 1–7 (to capture the early portion of learning, before reaching asymptotic performance) for the four ROIs identified by the DTI analysis (hippocampus, precuneus, posterior parietal cortex, and primary motor cortex), and compared it to a reference period. This reference corresponded to the final 30 seconds of the experiment, during which participants viewed a neutral fixation cross distinct from the task cues, signaling the conclusion of the task. We did not rely on the fMRI contrasts shown in Figure 2B of the manuscript, as they reflect relative differences between conditions and cannot directly address absolute levels of activation (Task > Rest and Rest > Task).

As depicted in Figure 1, all four regions showed activity above this reference period in both conditions (one-tailed t-test against zero; hippocampus Rest: $p < 0.001$, Task: $p = 0.004$; M1 Rest: $p = 0.003$, Task: $p < 0.001$; PPC Rest: $p = 0.004$, Task: $p < 0.001$; precuneus Rest: $p = 0.002$, Task: $p = 0.001$), indicating that changes in SANDI metrics occur within a functionally engaged network. Importantly, this overlap suggests that both changes in FSOMA and FNEURITE took place in brain regions that were metabolically active during learning. The transient and spatially widespread FSOMA increase is therefore consistent with activity-dependent processes linked to energy demand, such as ionic buffering (leading to swelling). In contrast, the more sustained and spatially restricted increase in FNEURITE likely reflects structural remodeling processes that evolve over a longer timescale. We cannot speculate about the relationship between FNEURITE and the BOLD signal beyond its anatomical overlap.

We now mention the functional/structural overlap in the revised manuscript (Results section: "SANDI uncovers distinct cellular contributions to MD dynamics", second paragraph), and in more detail in the Supporting Information. The corresponding methodology has also been added to the Methods section (Data analysis and statistics, Functional MRI, second paragraph).

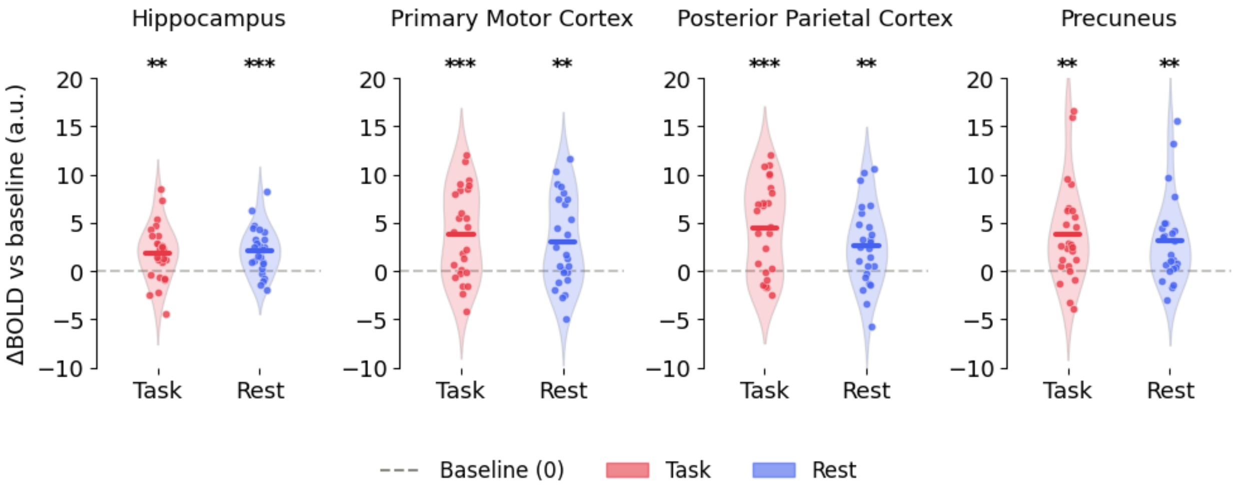

Figure 1. Functional activation during early learning. Violin plots show the mean change in BOLD signal relative to a resting-state baseline, for each of the four ROIs identified by the DTI analysis (hippocampus, M1, PPC, and precuneus) during Task (red) and Rest (blue) conditions across the first 7 blocks of practice (early learning). Dots represent individual participants; horizontal bars indicate the group mean; dashed lines indicate the baseline. Asterisks represent significant activation above baseline (one-sample t-test against zero; **p < 0.01, ***p < 0.001). Note that there may be some differences from the whole-brain analysis given that only early changes are considered herein.

3. It was hard to understand what was meant by "homeostatic changes". This phrase is repeatedly used; however, it was not clear to me how the changes related to homeostasis. Learning is the perturbing event, and there is then a process to return the biological system to a set-point (homeostasis), which in the case of say blood loss (perturbing event) is vasoconstriction (homeostatic process) to achieve a consistent set point (blood pressure). Beyond learning being the perturbing event, I am confused and unclear about all the other aspects of homeostasis (the process, the set point).

We thank the reviewer for pointing this out and agree that the link to homeostasis required a clearer explanation. In biology, homeostasis refers to the process by which biological systems maintain stable internal conditions despite perturbations through dynamic, reversible adjustments. Based on existing literature (Le Bihan et al., 2006; Jin et al., 2013; Mader et al., 2019), we hypothesize that the learning-related increases in neural activity described in the previous point likely led to membrane depolarization, transiently altering the ionic balance of the interstitial space. This triggered compensatory responses that buffered the ionic imbalance through cellular uptake, with water flowing osmotically into the cells leading to swelling (Le Bihan et al., 2006; Jin et al., 2013; Abe et al., 2017; Mader et al., 2019). The increase in FSOMA at 30 minutes is consistent with this interpretation, while its return to baseline at 24 hours is consistent with the restoration of ionic balance toward physiological levels — confirming the reversibility of the process.

Framed in the reviewer's own terms: the perturbing event is the sustained neural activity and membrane depolarization associated with learning; the set point corresponds to physiological ionic and electrochemical balance; the homeostatic process is ionic

buffering through cellular uptake; and the dynamic, reversible adjustment is the transient cellular swelling that resolves once ionic balance is restored, as reflected in the return of FSOMA to baseline at 24 hours.

We have clarified this interpretation in the revised manuscript (Results section: "SANDI uncovers distinct cellular contributions to MD dynamics", second paragraph).

4. The relationship between changes interpreted as cell body changes (FSOMA) and those that are interpreted as cell process (FNEURITE) changes could be revealing and strengthen the perspective provided in the work. Specifically, the cell body increasing in size could be due to protein synthesis required for distal cell process changes (FNEURITE). Could those brain areas (precuneus and parietal) showing lasting changes have cell bodies in those brain areas showing short term changes (FSOMA)?

We thank the reviewer for this interesting suggestion. It is indeed possible that the biological processes associated with FSOMA and FNEURITE are functionally related. Consistent with this possibility, we observe an anatomical overlap between transient FSOMA changes and persistent FNEURITE changes in the precuneus and PPC, suggesting that the early activity-dependent response may influence subsequent structural remodeling in these regions. However, directly testing this relationship through correlation is not appropriate since FSOMA, FNEURITE, and FEXTRA are compositionally dependent (together with FEXTRA they sum to one), precluding a meaningful interpretation of any observed correlation between them. This is a limitation of the model that we address in the revised manuscript (Discussion, sixth paragraph).

We note, however, that the two processes can be dissociated — hippocampus and M1 show transient FSOMA changes without any corresponding FNEURITE increase — suggesting that activity-dependent swelling does not necessarily lead to structural remodeling in all engaged regions. Addressing this issue would require experimental approaches in non-human animals with higher temporal resolution and direct cellular validation.

5.It was also not clear to me how this work fits in or alters our understanding of offline memory processing? Is the long-term storage of a sequence not within the hippocampus but in the cortex (such as the parietal cortex)? Is this what happens due to consolidation, and perhaps sleep?

Our findings can be interpreted within the framework of systems consolidation, whereby the hippocampus plays a time-limited role in early memory encoding and contributes to the emergence of longer-term representations in distributed cortical regions.

In our data, the hippocampus was more active during rest periods interleaved with practice (Figure 2B of the manuscript), consistent with memory reactivation — a phenomenon supported by recent evidence for hippocampal replay during waking rest in humans (Buch et al., 2021; Sjøgård et al., 2025), and previously linked to micro-offline gains in our earlier work (Jacobacci et al., 2020a). Consistent with this functional engagement, the hippocampus also shows a transient increase in FSOMA at 30 minutes, with no corresponding change in FNEURITE, suggesting that its involvement at this stage reflects

activity-dependent processes associated with early encoding and reactivation, rather than lasting structural remodeling. In contrast, the precuneus and PPC exhibit persistent increases in FNEURITE, consistent with the emergence of longer-term cortical representations.

Importantly, the increment in FNEURITE at 30 minutes suggests that cortical structural remodeling is initiated rapidly after learning, rather than being exclusively dependent on offline processes such as sleep (Paired t-test FNEURITE 30 minutes vs baseline; PPC: $T(27)=3.67$, $p=0.0010$; precuneus: $T(27)=3.48$, $p=0.0017$). This is in line with previous work from our team and others indicating that hippocampal–cortical interactions begin immediately after learning (Brodt et al., 2018; Jacobacci et al., 2020a), suggesting a gradual process that can be initiated early after learning independently of sleep.

We note, however, that this interpretation is specific to a single session of learning. More intensive training or differences in task structure, such as the degree of implicit versus explicit learning, may lead to longer-lasting hippocampal microstructural changes, as suggested by recent work from our group showing that both the magnitude and persistence of mean diffusivity changes are modulated by the implicit/explicit nature of the task (Griffa et al., 2025).

While our design does not allow us to directly assess the role of sleep, it remains possible that sleep contributes to the further stabilization of structural remodeling initiated during waking. We have clarified this interpretation in the revised manuscript (Discussion, fourth paragraph)

6. Given that sleep is meant to decrease synaptic density (synaptic downscaling) would there not have been an expected decrease in the FNEURITE signal (broadly) across many neural circuits? Is it possible to account for this apparent discrepancy between these results. I suspect it comes down to baseline and when it was taken relative to sleep. However, I think it might be nice to make this issue explicit so that other readers do not need to think this through for themselves.

We thank the reviewer for raising this important point. As the reviewer correctly suspects, this comes down to the baseline and what our measure captures. Our measurements are relative to a pre-learning baseline acquired before any sleep had occurred, and therefore reflect the net outcome of learning-related changes and any sleep-dependent processes combined. More fundamentally, FNEURITE is sensitive to larger cellular processes such as axons and dendrites, and is unlikely to be sensitive to the fine synaptic changes predicted by the synaptic homeostasis hypothesis under our current acquisition parameters (Christiaens et al., 2022). Whether synaptic downscaling occurred during sleep therefore cannot be addressed from our data, and the sustained FNEURITE increase at 24h does not argue for or against it. We have clarified this point in the Discussion (fourth paragraph).

7. How many of these changes are perhaps due to performance of a sequence as opposed to learning a sequence? Would the human brain be unaffected by making repeated movements without any serial structure?

We thank the reviewer for raising this important point. The regional specificity and temporal dynamics of the observed effects support a learning-related interpretation: sustained FNEURITE increases were restricted to the precuneus and PPC — regions implicated in motor planning and sequence learning (Karni et al., 1998; Doyon et al., 2003) — and were positively correlated with micro-offline gains, linking these microstructural changes to behavioral indices of learning-specific consolidation (see response to Reviewer 3, Point 1).

We acknowledge, however, that fully dissociating the effects of serial structure from learning remains an open question. Even without explicit learning, sequential structure may engage processes such as statistical learning and temporal prediction (Kornysheva et al., 2019; Ariani & Diedrichsen, 2019; Yewbrey & Kornysheva, 2024), and isolating their contribution remains an important challenge for future work.

Minor/Specific comments:

On page 3, the sentence starting "In sum, these….." I did not understand how this related to the earlier paragraph that seemed to discuss learning as opposed to performance.

We have rephrased this sentence to more clearly reflect the preceding paragraph: "In sum, these results replicate our previous behavioral, fMRI, and DTI findings obtained from a study comparing a learning group to a non-learning control group performing repeated, non-sequential finger tapping (Jacobacci et al., 2020a). This replication provides support for the learning specificity of the microstructural changes reported here and strengthens the basis for their cellular-level interpretation" (Results, Third paragraph).

On page 7, I didn't understand the value provided by this analysis. I am sure it is valuable. I think it could be just better articulated how and what it adds.

We thank the reviewer for pointing this out. The value of this analysis lies in addressing a circularity concern inherent to our main analytical approach. Because SANDI metrics were extracted within DTI-defined clusters, one could reasonably question whether the SANDI results are genuinely independent of the DTI results or whether they simply reflect the same signal repackaged through a different model. Figure 4 of the manuscript directly addresses this by applying DTI and SANDI fully independently across the six hippocampal subfields. The remarkable spatial and temporal correspondence between MD and FSOMA across subfields confirms convergent validity between the two models and demonstrates that the compartment-specific changes detected by SANDI are not an artifact of having been applied within DTI-defined regions. The hippocampal subfields were chosen specifically because they represent a tractable number of independently defined anatomical regions, avoiding the multiple comparisons problem that would arise from parcellating the entire cortex and subcortex (>130 ROIs).

We have revised the manuscript to better articulate this rationale (Results Section: "Independent, cross-model validation of SANDI", first paragraph).

Reviewer #3: The manuscript by Griffa et al. investigates neuroplasticity in response to motor learning using novel diffusion MRI methods. The main aim is to overcome previous limitations of conventional diffusion MRI methods in capturing biologically specific brain changes in humans. Longitudinal studies have started to investigated learning-induced neuroplasticity in humans approx. 20 years ago. Diffusion MRI is sensitive to neuroplasticity in grey matter regions and several studies over the past 10 years have consistently shown motor learning-related changes in hippocampal networks using diffusion MRI. The current study leverages the specificity of new MRI hardware (connectome MRI scanner) and software (SANDI) to cell-specific grey matter changes (e.g., astrocytes, neurons). SANDI is one of many new methods proposing increased biological specificity of parameters derived from in vivo diffusion MRI data. Griffa et al. obtained three diffusion MRI scans per participant:before, 30 min. as well as 24h after the first practice session of a new finger sequence task. Results show short-term and transient increases in a cell-specific SANDI marker in hippocampus and motor cortex as well as short-term but persistent (remained at least until 24h) neurite-specific changes in posterior parietal cortex and precuneus. The authors conclude that the results differentiate between plastic (persistent) and non-plastic (transient) changes and provide a mechanistic window into human neuroplasticity relevant to bridge the gap between human and animal neuroscience research. The evidence is based on (1) a well-established learning paradigm (finger sequence learning) suitable for application within the MR-environment, (2) a beneficial combination of diffusion-based MRI and task-based fMRI for localizing relevant brain areas and (3) a remarkable consistency of previous findings (partly from the same group) on hippocampal involvement in early motor sequence
learning using conventional DTI methods.

Overall, the results appear in line with previous DTI findings and the new methods (SANDI, connectome MR scanner) enabled the discovery of dissociable temporal dynamics in presumably cell- and neurite-specific brain changes underlying the DTI signal - which is indeed a leap forward for the human imaging field and a stimulus for new hypotheses testable with cross-species animal and human MRI studies.

We thank the reviewer for the constructive feedback.

However, there are major concerns related to the validity of conclusions which I describe below:

1. The authors did not include a control group that would allow them to relate SANDI changes to learning (rather than unspecific changes over time). This is unusual, in part, because the sequence learning paradigm has been used for decades using well-controlled study designs. The authors mentioned the consistency of DTI- and fMRI findings with their previous work (that included a non-learning control group), but due to the lack of additional correlations between SANDI changes and learning parameters, the nature of cell- and neurite-specific changes remain elusive. The lack of behavioral correlations is unexpected given the wealth of learning parameters captured by the study design (micro-offline gains, micro-online gains, retention performance…). A proper control group or correlations with learning parameters would be essential to associate brain changes to behavior.

We thank the reviewer for pointing this out. We acknowledge that the replication of DTI and fMRI findings from our previous study including a non-learning control group (Jacobacci et al., 2020a) provides only indirect support for the learning specificity of the SANDI changes reported here. To directly examine the relationship between the time course of FSOMA and FNEURITE and behavioral measures of learning, we conducted hypothesis-driven linear mixed-effects models, with timepoint (30 min, 24 h) and the behavioral measure as fixed effects, and subject included as a random intercept to account for repeated measures (LMMs; see Methods: Data Analysis and Statistics, Diffusion MRI). For FSOMA, the key term of interest was the timepoint × behavioral metric interaction, consistent with a transient response. For FNEURITE, the key term was the main effect of the behavioral metric, consistent with a fast but more persistent structural change.

The analysis revealed a significant association between the time course of FNEURITE and micro-offline gains ($F(1,25) = 6.15$, $p = 0.020$). The regression coefficients for FNEURITE at each timepoint indicate a positive relationship at both 30 min and 24h (β 30min = 0.007, β 24h = 0.02), which was significantly stronger at 24h than at 30 min (timepoint × MOG interaction: $F(1,79) = 3.95$, $p = 0.05$). This suggests that participants showing greater early consolidation during learning exhibited larger and more persistent increases in cell-process density, consistent with structural remodeling.

Taken together, the significant association between FNEURITE and micro-offline gains provides evidence linking structural remodeling to early consolidation, pointing to a genuine learning-related process rather than unspecific changes over time. We note, however, that relationships between SANDI metrics and learning/retention may exist that are not captured by the present linear approach, as microstructural changes induced by motor learning may follow nonlinear dynamics (Xu et al., 2009; Yang et al., 2009; Hille et al., 2024). These findings have now been incorporated into the revised manuscript (Results section: "*SANDI uncovers distinct cellular contributions to MD dynamics*", fourth paragraph).

2. With respect to 1., I think that the evidence for concluding "distinct plastic and non-plastic changes" underlying the SANDI signals is low due to the following reasons: first, there are lots of relevant microstructural changes induced during initial skill learning and MRI may only capture the tip of the iceberg. Theory suggests non-linear brain changes during motor skill acquisition (see Hille et al., 2024). Labelling a transient change (fig. 3A) as "non-plastic" ignores the large number of ongoing changes causally relevant for learning (e.g., the clustered addition and pruning of new synaptic spines) but potentially not detectable with diffusion MRI.

We thank the reviewer for this important point. We would like to clarify what we mean by "non-plastic" in the context of FSOMA. We are not claiming that plastic processes are absent during early learning — we fully acknowledge that such processes are likely occurring but fall below the detection threshold of our current acquisition parameters. What we label as non-plastic refers exclusively to the reversibility of what FSOMA detects: a transient volumetric increment occurring shortly after learning that returns to baseline overnight. By no means do we imply that this homeostatic response is biologically irrelevant or does not contribute to neuroplasticity.

Interestingly, two of the four regions showing transient FSOMA increases — precuneus and PPC — are the same regions that also exhibit lasting FNEURITE increases, raising the possibility that the two processes are linked. Due to the compositional structure of the SANDI model, however, we cannot directly test this relationship. We have added a sentence to the Results section to clarify both points (Results: "SANDI uncovers distinct cellular contributions to MD dynamics", last paragraph).

Second, dissociable time courses should be inferred from direct statistical comparisons of SANDI changes (e.g., using analyses of variance) rather than separate region-wise analyses (as done throughout the manuscript).

We agree that it would be ideal to examine SANDI metrics across the whole brain. However, SANDI compartment fractions are compositional variables: FSOMA, FNEURITE, and FEXTRA are constrained to sum to one within each voxel, leading to statistical dependence among them. This is a limitation of the model that we address in the Discussion (sixth paragraph of the Discussion section). As a result, running wholebrain voxel-wise separate ANOVAs on individual fractions does not provide an independent test of dissociable effects, since increases in one compartment fraction may imply decreases in the other.

Focusing on SANDI changes within the four regions identified from the whole-brain DTI analysis allowed us to directly probe the cellular basis of learning-related changes driving MD, while limiting the analysis to a defined set of regions and avoiding an inflation of multiple comparisons.

Finally, note that the analysis in Figure 4 was specifically designed to address the concern of circularity inherent in fitting SANDI within DTI-defined regions. Specifically, DTI and SANDI were applied independently across anatomically defined hippocampal subfields, providing an unbiased test of cross-model correspondence. The shared topography and temporal dynamics between MD and FSOMA across subfields confirm convergent validity of the SANDI approach, while keeping the number of comparisons tractable.

3. The authors interpret the increase in soma fraction as potential correlate of activity-induced cell swelling. In the abstract, they note that "We found that learning a motor skill triggered two distinct temporal responses: a transient expansion of cell bodies across all brain regions engaged by the task…". Right-handed participants practiced the finger sequence using their non-dominant left hand and fMRI results indicate task-induced activity in the contralateral right M1 (fig. 2B). However, FSOMA changes appeared in left but not right M1 (figs. 2, 3) which argues against brain activity as driver of FSOMA changes. In addition to that, the hippocampus was more active in the resting periods between practice blocks compared to the actual task execution (fig. 2). It is not clear, however, if this is the results of a deactivation during practice blocks or an activation during rest. Together, the relation between brain activity and cell-specific transient change remains unclear.

We thank the reviewer for pointing this out. The fMRI contrasts shown in Figure 2B of the manuscript reflect relative differences between conditions (Task > Rest and Rest > Task)

and therefore do not allow us to determine whether a given region was active per se during Task or Rest periods.

To address this question, we extracted the mean BOLD signal across blocks 1–7 ((to capture the early portion of learning, before reaching asymptotic performance) during Task and Rest, for the four ROIs showing significant FSOMA changes, and compared each condition to a reference period. This period corresponded to the final 30 seconds of a 1-minute interval at the end of the experiment, which signaled the conclusion of the task, during which participants were instructed to remain still while viewing a neutral fixation cross distinct from the task cues. As shown in Figure 1 (Point # 2 of Reviewer 2), all ROIs showed significant activity above this reference in both conditions (one-tailed t-test; hippocampus Rest: $p < 0.001$, Task: $p = 0.004$; M1 Rest: $p = 0.003$, Task: $p < 0.001$; PPC Rest: $p = 0.004$, Task: $p < 0.001$; precuneus Rest: $p = 0.002$, Task: $p = 0.001$), confirming that all regions were genuinely engaged during learning.

Regarding the lateralization of microstructural changes to the left hemisphere, this spatial pattern has been observed across motor learning paradigms regardless of the trained hand (Jacobacci et al., 2020a; Griffa et al., 2025), and is consistent with the well-established left-lateralization of motor planning for skilled movements (De Renzi & Lucchelli, 1988; Schaefer et al., 2007; Yang et al., 2024; Merrick et al., 2022).

These results have been incorporated into the revised manuscript (Results: second paragraph of "Sandi uncovers…" section; Methods: Functional MRI, second paragraph; Supporting Information).

4. SANDI offers readouts of cell diameter in addition to the fractions of soma, neurite and free water. Cell diameter was not analysed in the current study but this parameter would be more specific to the proposed cell swelling mechanism (rather than soma fraction).

We thank the reviewer for this suggestion. We note, however, that the reliable estimation of apparent soma radius (RSOMA) in vivo is recognized as a significant technical challenge, even under optimal acquisition conditions. As explicitly acknowledged by the developers of the SANDI model, RSOMA estimates lack accuracy and precision when derived from data acquired with a limited number of b-values and a single diffusion time, as used in the Connectome scanner for this study (Palombo et al., 2020). Given this limitation, we focused on FSOMA, which is the more robust and interpretable metric for characterizing changes in cell bodies under our acquisition conditions.

5. The empirical basis of cell-specific (rather than neurite-specific) plasticity through short-term learning remains to be shown in the intro. Cited studies largely reflect neurite-specific mechanisms but then, why was SANDI and not another diffusion model used in the context of this study?

The present study was directly motivated by our previous work (Jacobacci et al., 2020a), in which we observed learning-induced changes in mean diffusivity (MD) that differed in their temporal dynamics across brain regions. Given that MD reflects a combination of intra- and extracellular water diffusion, it cannot by itself identify which cellular compartment was driving the observed changes, nor whether these reflect neuroplasticity

and/or non-specific processes such as ionic buffering. We chose SANDI to separately estimate soma- and neurite-related signal contributions in gray matter, allowing us to directly address this ambiguity.

6. Please also describe the evidence from histological validation studies indicating cell-specific mechanisms as a correlate of SANDI changes.

We thank the reviewer for this suggestion. Several lines of evidence support the biological interpretability of SANDI parameters. The original SANDI paper (Palombo et al., 2020) showed that soma and neurite fraction maps qualitatively reproduce cytoarchitectural profiles from histology, and subsequent work in the mouse brain demonstrated correspondence between SANDI-derived soma fractions and cell body density estimates from the Allen Brain Atlas (Ianuș et al., 2022). Preclinical studies in glioma models combining diffusion MRI with histopathology further show that SANDI parameters vary consistently with known differences in cell size and tissue organization (Ianuș et al., 2023; Palombo et al., 2021). In vivo, SANDI captures biologically meaningful variation including cortical laminar cyto-architecture consistent with histological literatures (Lee et al., 2026), age-related microstructural changes (Lee et al., 2024; Karat et al., 2026) and alterations in neurological conditions such as Alzheimer's disease and Multiple Sclerosis consistent with known pathological processes including neuronal loss and microstructural degeneration (Margoni et al., 2022; Ianuș et al., 2023; Krijnen et al., 2023). We have added the relevant references to the revised manuscript (Results section: "SANDI uncovers distinct cellular contributions to MD dynamics", first paragraph; Discussion, first paragraph).

REFERENCES

- Abe, Y., Van Nguyen, K., Tsurugizawa, T., Ciobanu, L., & Le Bihan, D. (2017). Modulation of water diffusion by activation-induced neural cell swelling in Aplysia Californica. *Scientific reports*, *7*(1), 6178. https://doi.org/10.1038/s41598-017-05586-5

- Ariani, G., & Diedrichsen, J. (2019). Sequence learning is driven by improvements in motor planning. Journal of neurophysiology, 121(6), 2088–2100. https://doi.org/10.1152/jn.00041.2019

- Ariani, G., Kordjazi, N., Pruszynski, J. A., & Diedrichsen, J. (2021). The Planning Horizon for Movement Sequences. eNeuro, 8(2), ENEURO.0085-21.2021. https://doi.org/10.1523/ENEURO.0085-21.2021

- Brodt, S., Gais, S., Beck, J., Erb, M., Scheffler, K., & Schönauer, M. (2018). Fast track to the neocortex: A memory engram in the posterior parietal cortex. Science (New York, N.Y.), 362(6418), 1045–1048. https://doi.org/10.1126/science.aau2528

- Buch, E. R., Claudino, L., Quentin, R., Bönstrup, M., & Cohen, L. G. (2021). Consolidation

of human skill linked to waking hippocampo-neocortical replay. Cell reports, 35(10), 109193. https://doi.org/10.1016/j.celrep.2021.109193

- Christiaens, D., Vande Casteele, T., Laroy, M., Van Cauwenberge, M., Van den Stock, J., Bouckaert, F., ... & Emsell, L. (2022, May). Correlating neurite density and synaptic density in the human brain in vivo with diffusion-weighted PET-MR. In Joint Annual Meeting ISMRM-ESMRMB, Date: 2022/05/07-2022/05/12, Location: London, UK.

- De Renzi, E., & Lucchelli, F. (1988). Ideational apraxia. Brain: a journal of neurology, 111 (Pt 5), 1173–1185. https://doi.org/10.1093/brain/111.5.1173

- Doyon, J., Penhune, V., & Ungerleider, L. G. (2003). Distinct contribution of the cortico-striatal and cortico-cerebellar systems to motor skill learning. *Neuropsychologia*, *41*(3), 252–262. https://doi.org/10.1016/s0028-3932(02)00158-6

- Griffa, G., Solano, A., Deleglise, A., De Pino, G., Jacobacci, F., & Della-Maggiore, V. (2025). Uncovering the Role of the Human Hippocampus in Procedural Motor Learning: Insights from Implicit Sensorimotor Adaptation. The Journal of neuroscience : the official journal of the Society for Neuroscience, 45(36), e2119242025. https://doi.org/10.1523/JNEUROSCI.2119-24.2025

- Hille, M., Kühn, S., Kempermann, G., Bonhoeffer, T., & Lindenberger, U. (2024). From animal models to human individuality: Integrative approaches to the study of brain plasticity. Neuron, 112(21), 3522–3541. https://doi.org/10.1016/j.neuron.2024.10.006

- Ianuş, A., Carvalho, J., Fernandes, F. F., Cruz, R., Chavarrias, C., Palombo, M., & Shemesh, N. (2022). Soma and Neurite Density MRI (SANDI) of the in-vivo mouse brain and comparison with the Allen Brain Atlas. *NeuroImage*, *254*, 119135. https://doi.org/10.1016/j.neuroimage.2022.119135

- Ianus, A., Palombo, M., Simoes, R. V., Henriques, R. N., Carvalho, T., & Shemesh, N. Soma and Neurite Density Imaging (SANDI) characterizes microscopic dìerences between two glioma mouse model subtypes (2023). Soma-and-Neurite-Density-Imaging-SANDI-characterizes-microscopic-differences-between-two-glioma-mouse-model-subtypes.pdf

- Jacobacci, F., Armony, J. L., Yeffal, A., Lerner, G., Amaro, E., Jr, Jovicich, J., Doyon, J., & Della-Maggiore, V. (2020a). Rapid hippocampal plasticity supports motor sequence learning. Proceedings of the National Academy of Sciences of the United States of America, 117(38), 23898–23903. https://doi.org/10.1073/pnas.2009576117

- Jin, B. J., Zhang, H., Binder, D. K., & Verkman, A. S. (2013). Aquaporin-4-dependent K(+) and water transport modeled in brain extracellular space following neuroexcitation. *The Journal of general physiology*, *141*(1), 119–132. https://doi.org/10.1085/jgp.201210883

- Karat, B. G., Genc, S., Raven, E. P., Palombo, M., Khan, A. R., & Jones, D. K. (2026). Microstructural variation of hippocampal substructures across childhood and adolescence quantified with high-gradient diffusion MRI. Communications biology, 9(1), 416. https://doi.org/10.1038/s42003-026-09622-x

- Karni, A., Meyer, G., Rey-Hipolito, C., Jezzard, P., Adams, M. M., Turner, R., & Ungerleider, L. G. (1998). The acquisition of skilled motor performance: fast and slow experience-driven changes in primary motor cortex. *Proceedings of the National Academy of Sciences of the United States of America*, *95*(3), 861–868. https://doi.org/10.1073/pnas.95.3.861

- Kornysheva, K., Bush, D., Meyer, S. S., Sadnicka, A., Barnes, G., & Burgess, N. (2019). Neural Competitive Queuing of Ordinal Structure Underlies Skilled Sequential Action. Neuron, 101(6), 1166–1180.e3. https://doi.org/10.1016/j.neuron.2019.01.018

- Krijnen, E. A., Russo, A. W., Salim Karam, E., Lee, H., Chiang, F. L., Schoonheim, M. M., Huang, S. Y., & Klawiter, E. C. (2023). Detection of grey matter microstructural substrates of neurodegeneration in multiple sclerosis. Brain communications, 5(3), fcad153. https://doi.org/10.1093/braincomms/fcad153

- Le Bihan, D., Urayama, S., Aso, T., Hanakawa, T., & Fukuyama, H. (2006). Direct and fast detection of neuronal activation in the human brain with diffusion MRI. Proceedings of the National Academy of Sciences of the United States of America, 103(21), 8263–8268. https://doi.org/10.1073/pnas.0600644103

- Lee, H., Lee, H. H., Ma, Y., Eskandarian, L., Gaudet, K., Tian, Q., Krijnen, E. A., Russo, A. W., Salat, D. H., Klawiter, E. C., & Huang, S. Y. (2024). Age-related alterations in human cortical microstructure across the lifespan: Insights from high-gradient diffusion MRI. *Aging cell*, e14267. Advance online publication. https://doi.org/10.1111/acel.14267

- Lee, H., Ma, Y., Chan, K. S., Krijnen, E. A., Eskandarian, L., Bhatt, A., Gerold, J., Mahmutovic, M., Puonti, O., Zeng, X., Deden Binder, L. J., Fischl, B., Keil, B., Ramos-Llordén, G., Klawiter, E. C., Lee, H. H., & Huang, S. Y. (2026). Visualizing cortical laminar architecture in the living human brain using next-generation ultra-high-gradient diffusion MRI. Communications biology, 10.1038/s42003-026-09887-2. Advance online publication. https://doi.org/10.1038/s42003-026-09887-2

- Mader, S., & Brimberg, L. (2019). Aquaporin-4 Water Channel in the Brain and Its Implication for Health and Disease. *Cells*, *8*(2), 90. https://doi.org/10.3390/cells8020090

- Margoni, M., Pagani, E., Preziosa, P., Palombo, M., Gueye, M., Azzimonti, M., Filippi, M., & Rocca, M. A. (2023). In vivo quantification of brain soma and neurite density abnormalities in multiple sclerosis. Journal of neurology, 270(1), 433–445. https://doi.org/10.1007/s00415-022-11386-3

- Merrick, C. M., Dixon, T. C., Breska, A., Lin, J., Chang, E. F., King-Stephens, D., Laxer, K. D., Weber, P. B., Carmena, J., Thomas Knight, R., & Ivry, R. B. (2022). Left hemisphere dominance for bilateral kinematic encoding in the human brain. eLife, 11, e69977. https://doi.org/10.7554/eLife.69977

- Palombo, M., Ianus, A., Guerreri, M., Nunes, D., Alexander, D. C., Shemesh, N., & Zhang, H. (2020). SANDI: A compartment-based model for non-invasive apparent soma and neurite imaging by diffusion MRI. *NeuroImage, 215,* 116835. https://doi.org/10.1016/j.neuroimage.2020.116835

- Palombo, M., Alexander, D. C., & Zhang, H. (2021). Large-scale analysis of brain cell morphometry informs microstructure modelling of gray matter. In Proceedings of the 2021 ISMRM & SMRT Annual Meeting and Exhibition, Virtual Meeting. Large-scale-analysis-of-brain-cell-morphometry-informs-microstructure-modelling-of-gray-matter.pdf

- Sagi, Y., Tavor, I., Hofstetter, S., Tzur-Moryosef, S., Blumenfeld-Katzir, T., & Assaf, Y. (2012). Learning in the fast lane: new insights into neuroplasticity. Neuron, 73(6), 1195–1203. https://doi.org/10.1016/j.neuron.2012.01.025

- Schaefer, S. Y., Haaland, K. Y., & Sainburg, R. L. (2007). Ipsilesional motor deficits following stroke reflect hemispheric specializations for movement control. Brain: a journal of neurology, 130(Pt 8), 2146–2158. https://doi.org/10.1093/brain/awm145

- Sjøgård, M., Baxter, B., Mylonas, D., Thompson, M., Kwok, K., Driscoll, B., Tolosa, A., Shi, W., Stickgold, R., Vangel, M., Chu, C. J., & Manoach, D. S. (2025). Hippocampal ripples predict motor learning during brief rest breaks in humans. Nature communications, 16(1), 6089. https://doi.org/10.1038/s41467-025-61136-y

- Xu, T., Yu, X., Perlik, A. J., Tobin, W. F., Zweig, J. A., Tennant, K., Jones, T., & Zuo, Y. (2009). Rapid formation and selective stabilization of synapses for enduring motor memories. Nature, 462(7275), 915–919. https://doi.org/10.1038/nature08389

- Yang, G., Pan, F., & Gan, W. B. (2009). Stably maintained dendritic spines are associated with lifelong memories. Nature, 462(7275), 920–924. https://doi.org/10.1038/nature08577

- Yang 杨炀, Y., Li 李君君, J., Zhao 赵恺, K., Tam, F., Graham, S. J., Xu 徐敏, M., & Zhou 周可, K. (2024). Lateralized Functional Connectivity of the Sensorimotor Cortex and its Variations During Complex Visuomotor Tasks. The Journal of neuroscience: the official journal of the Society for Neuroscience, 44(5), e0723232023. https://doi.org/10.1523/JNEUROSCI.0723-23.2023

- Yewbrey, R., & Kornysheva, K. (2024). The Hippocampus Preorders Movements for Skilled Action Sequences. The Journal of neuroscience : the official journal of the Society for Neuroscience, 44(45), e0832242024. https://doi.org/10.1523/JNEUROSCI.0832-24.2024
