# Supplementary material for: Learning engages transient and sustained cellular mechanisms in the human brain
Source: PLoS Biol. 2026 Jun 18;24(6):e3003861. doi: 10.1371/journal.pbio.3003861 (PMC13298990; doi:10.1371/journal.pbio.3003861)
Supplement: Attachment — Submitted filename: Authors Response.pdf [file pbio.3003861.s006.pdf]

Reviewer #3: Thank you very much for your responses to my concerns. The manuscript improved significantly. The important behavioral correlations suggest a link between diffusion changes and persistent improvements in performance. In your response to point 3, the lateralization issue was not sufficiently addressed in my opinion. I got the point that left-hand practice engages contralateral AND ipsilateral M1 (consistent with ealier studies), but this does not explain why the highly active right M1 was not affected by diffusion changes. This seems inconsistent with the interpretation that practice-induced increases in brain activity drive neural plasticity. Please provide an explanation for this result.

We thank the reviewer for pressing on this point. The magnitude of the BOLD response does not reflect the underlying neuronal process driving motor learning or movement execution. An increase in fMRI activity is therefore a necessary but not sufficient condition to infer neuroplasticity. We believe that the absence of diffusion changes in right M1 is thus not inconsistent with the activity-dependent hypothesis, but instead suggests that the functional role of a brain region within the motor network - rather than the magnitude of its BOLD response alone - determines whether learning-related neuroplasticity occurs.
