# Supplementary material for: Learning engages transient and sustained cellular mechanisms in the human brain
Source: PLoS Biol. 2026 Jun 18;24(6):e3003861. doi: 10.1371/journal.pbio.3003861 (PMC13298990; doi:10.1371/journal.pbio.3003861)
Supplement: Peer review history

# Editor report: Decision Letter 0

**DOI:** 10.1371/journal.pbio.3003861.r001

**Authors:** Christian Schnell, PhD

19 Dec 2025

Dear Valeria,

Thank you for submitting your manuscript entitled "A mechanistic window onto human neuroplasticity through cell-specific diffusion MRI" for consideration as a Short Reports by PLOS Biology.

Your manuscript has now been evaluated by the PLOS Biology editorial staff and I am writing to let you know that we would like to send your submission out for external peer review. I should note, however, in the absence of advice from one of our academic editors, that we were not fully convinced whether the conceptual advance is sufficient for us. We will be discussing this with the reviewers and the academic editor after review.

However, before we can send your manuscript to reviewers, we need you to complete your submission by providing the metadata that is required for full assessment. To this end, please login to Editorial Manager where you will find the paper in the 'Submissions Needing Revisions' folder on your homepage. Please click 'Revise Submission' from the Action Links and complete all additional questions in the submission questionnaire.

Once your full submission is complete, your paper will undergo a series of checks in preparation for peer review. After your manuscript has passed the checks it will be sent out for review. To provide the metadata for your submission, please Login to Editorial Manager (https://www.editorialmanager.com/pbiology) within two weeks, i.e. by Jan 01 2026 11:59PM.

Please note that our office will be closed starting on Monday for two weeks, so we will only be able to start inviting reviewers in the second week of January.

If your manuscript has been previously peer-reviewed at another journal, PLOS Biology is willing to work with those reviews in order to avoid re-starting the process. Submission of the previous reviews is entirely optional and our ability to use them effectively will depend on the willingness of the previous journal to confirm the content of the reports and share the reviewer identities. Please note that we reserve the right to invite additional reviewers if we consider that additional/independent reviewers are needed, although we aim to avoid this as far as possible. In our experience, working with previous reviews does save time.

If you would like us to consider previous reviewer reports, please edit your cover letter to let us know and include the name of the journal where the work was previously considered and the manuscript ID it was given. In addition, please upload a response to the reviews as a 'Prior Peer Review' file type, which should include the reports in full and a point-by-point reply detailing how you have or plan to address the reviewers' concerns.

During the process of completing your manuscript submission, you will be invited to opt-in to posting your pre-review manuscript as a bioRxiv preprint. Visit http://journals.plos.org/plosbiology/s/preprints for full details. If you consent to posting your current manuscript as a preprint, please upload a single Preprint PDF.

Feel free to email us at plosbiology@plos.org if you have any queries relating to your submission.

Kind regards,

Christian

Christian Schnell, PhD

Senior Editor

PLOS Biology

cschnell@plos.org

# Peer review: Decision Letter 1

**DOI:** 10.1371/journal.pbio.3003861.r002

**Authors:** Christian Schnell, PhD, Christian Schnell, PhD

13 Feb 2026

Dear Valeria,

Thank you for your patience while your manuscript "A mechanistic window onto human neuroplasticity through cell-specific diffusion MRI" was peer-reviewed at PLOS Biology. It has now been evaluated by the PLOS Biology editors, an Academic Editor with relevant expertise, and by several independent reviewers.

In light of the reviews, which you will find at the end of this email, we would like to invite you to revise the work to thoroughly address the reviewers' reports.

As you will see below, the reviewers find the topic of your study very interesting and are overall positive. However, the reviewers raise a number of important concerns that need to be addressed. While we ask you to address all concerns, we specifically we ask you to focus on (i) sharpening the scope and describe the advance of the study in more clarity and (ii) guiding the reader more clearly about what is being shown, how it is learning related, how we can be certain that these are structural changes, and that they relate to some aspect of learning. We think you will be able to address these concerns with textual revisions and (re)analyzing current data, and do not expect you to collect new experimental data.

However, these requests are not simply cosmetic changes. You will see in the reviewer reports that there is some ambiguity in how the reviewers view the advance of you study, so it needs to be very clear what the advance is, and that we can reliably attribute the DTI/SANDI changes to structural change.

Given the extent of revision needed, we cannot make a decision about publication until we have seen the revised manuscript and your response to the reviewers' comments. Your revised manuscript is likely to be sent for further evaluation by all or a subset of the reviewers.

In addition to these revisions, you will need to complete some formatting changes, which you will receive in a follow up email. A member of our team will be in touch with a set of requests shortly.

We expect to receive your revised manuscript within 3 months. Please email us (plosbiology@plos.org) if you have any questions or concerns, or would like to request an extension.

At this stage, your manuscript remains formally under active consideration at our journal; please notify us by email if you do not intend to submit a revision so that we may withdraw it.

**IMPORTANT - SUBMITTING YOUR REVISION**

Your revisions should address the specific points made by each reviewer. Please submit the following files along with your revised manuscript:

1. A 'Response to Reviewers' file - this should detail your responses to the editorial requests, present a point-by-point response to all of the reviewers' comments, and indicate the changes made to the manuscript.

*NOTE: In your point-by-point response to the reviewers, please provide the full context of each review. Do not selectively quote paragraphs or sentences to reply to. The entire set of reviewer comments should be present in full and each specific point should be responded to individually, point by point.

You should also cite any additional relevant literature that has been published since the original submission and mention any additional citations in your response.

2. In addition to a clean copy of the manuscript, please also upload a 'track-changes' version of your manuscript that specifies the edits made. This should be uploaded as a "Revised Article with Changes Highlighted" file type.

*Re-submission Checklist*

When you are ready to resubmit your revised manuscript, please refer to this re-submission checklist: https://plos.io/Biology_Checklist

To submit a revised version of your manuscript, please go to https://www.editorialmanager.com/pbiology/ and log in as an Author. Click the link labelled 'Submissions Needing Revision' where you will find your submission record.

Please make sure to read the following important policies and guidelines while preparing your revision:

*Published Peer Review*

Please note while forming your response, if your article is accepted, you may have the opportunity to make the peer review history publicly available. The record will include editor decision letters (with reviews) and your responses to reviewer comments. If eligible, we will contact you to opt in or out. Please see here for more details:

https://blogs.plos.org/plos/2019/05/plos-journals-now-open-for-published-peer-review/

*PLOS Data Policy*

Please note that as a condition of publication PLOS' data policy (http://journals.plos.org/plosbiology/s/data-availability) requires that you make available all data used to draw the conclusions arrived at in your manuscript. If you have not already done so, you must include any data used in your manuscript either in appropriate repositories, within the body of the manuscript, or as supporting information (N.B. this includes any numerical values that were used to generate graphs, histograms etc.). For an example see here: http://www.plosbiology.org/article/info%3Adoi%2F10.1371%2Fjournal.pbio.1001908#s5

*Blot and Gel Data Policy*

We require the original, uncropped and minimally adjusted images supporting all blot and gel results reported in an article's figures or Supporting Information files. We will require these files before a manuscript can be accepted so please prepare them now, if you have not already uploaded them. Please carefully read our guidelines for how to prepare and upload this data: https://journals.plos.org/plosbiology/s/figures#loc-blot-and-gel-reporting-requirements

*Protocols deposition*

To enhance the reproducibility of your results, we recommend that if applicable you deposit your laboratory protocols in protocols.io, where a protocol can be assigned its own identifier (DOI) such that it can be cited independently in the future. Additionally, PLOS ONE offers an option for publishing peer-reviewed Lab Protocol articles, which describe protocols hosted on protocols.io. Read more information on sharing protocols at https://plos.org/protocols?utm_medium=editorial-email&utm_source=authorletters&utm_campaign=protocols

Thank you again for your submission to our journal. We hope that our editorial process has been constructive thus far, and we welcome your feedback at any time. Please don't hesitate to contact us if you have any questions or comments.

Sincerely,

Christian

Christian Schnell, PhD

Senior Editor

PLOS Biology

cschnell@plos.org

------------------------------------

REVIEWS:

Reviewer #1 (Yaniv Assaf): This is a strong and conceptually important manuscript that makes a clear advance in human neuroplasticity research. By combining ultra-high-gradient diffusion MRI with the SANDI model, the authors provide one of the first in vivo demonstrations that learning-related microstructural changes in the human brain can be temporally and compartmentally dissociated into transient, non-plastic processes (cell-body expansion) and sustained, region-specific structural plasticity (increased cell-process density). This directly addresses a long-standing limitation of diffusion MRI studies, which have been unable to distinguish swelling or homeostatic responses from genuine remodeling.

Methodologically, the work is rigorous and well controlled. The longitudinal design, replication of prior behavioral/fMRI/DTI effects, use of ultra-high-gradient data, and convergent validation via hippocampal subfield analyses substantially strengthen confidence in the findings. The interpretation is biologically grounded, well integrated with animal literature, and avoids overstatement. Importantly, the results move the field beyond descriptive diffusion changes toward mechanistic inference, which is particularly valuable for studies of learning, development, and disease.

The main limitations (model assumptions, relative—not absolute—compartment fractions, and reliance on a single learning paradigm) are appropriately acknowledged and do not undermine the central conclusions. Any remaining issues are largely matters of framing and clarification, particularly with respect to the biological specificity of the model and the extent to which these findings can be generalized beyond the present task and acquisition regime. These do not represent conceptual or technical weaknesses of the study.

Overall, this is a high-quality and high-impact contribution that is very well aligned with the scope and standards of PLOS Biology. I therefore strongly recommend acceptance, with at most minor revisions focused on sharpening the discussion and boundaries of interpretation, rather than on additional analyses or changes to the core conclusions.

Reviewer #2: This is an interesting and fun manuscript to read. It makes a valuable contribution by showing that different aspects of an MRI (diffusion) signal can provide insight into different cellular processes, which are differentially affected by learning. It is a nice mix of technical development (validation) along with more cognitive/biological issues. Many interesting questions arise from these insights that I don't articulate here because they are beyond the scope of the current work; nonetheless, they speak to the importance (and potential impact) of the work.

There are several aspects that I struggled to understand, and so the work could benefit from clarification. These center around two major issues: (a) the relationship between the recorded signal and cellular changes; (b) new insights provided into motor memory processing. The following questions sometimes deal with one or other of these questions or at times are a bit overlapping.

Perhaps the greatest support for the two signals (FSOMA and FNEURITE) tapping into different biological processes is that they dissociate across time (and space). The FNEURITE signal as would be expected last longer (present at 24 hours) and for specific brain areas. It should be possible to highlight this important strength more in the work. Whilst the logic that these signals are tapping into different biological processes is sound there is not much evidence in the study that they are --- other than this very nice temporal dissociation.

What is the relationship between the functional activation (see Fig 2 - having these was a nice strength to the work) and the FNEURITE and FSOMA signals? Are they overlapping, and so come about due to energetic changes, and therefore consistent with the (energetic) cellular changes that are thought to be the basis for the different (MRI) signal changes?

It was hard to understand by what was meant by "homeostatic changes". This phrase is repeatedly used; however, it was not clear to me how the changes related to homeostasis. Learning is the perturbing event, and there is then a process to return the biological system to a set-point (homeostasis), which in the case of say blood loss (perturbing event) is vasoconstriction (homeostatic process) to achieve a consistent set point (blood pressure). Beyond learning being the perturbing event, I am confused and unclear about all the other aspects of homeostasis (the process, the set point).

The relationship between changes interpreted as cell body changes (FSOMA) and those that are interpreted as cell process (FNEURITE) changes could be revealing and strengthen the perspective provided in the work. Specifically, the cell body increasing in size could be due to protein synthesis required for distal cell process changes (FNEURITE). Could those brain areas (precuneus and parietal) showing lasting changes have cell bodies in those brain areas showing short term changes (FSOMA)?

It was also not clear to me how this work fits in or alters our understanding of offline memory processing? Is the long-term storage of a sequence not within the hippocampus but in the cortex (such as the parietal cortex)? Is this what happens due to consolidation, and perhaps sleep?

Given that sleep is meant to decrease synaptic density (synaptic downscaling) would there not have been an expected decrease in the FNEURITE signal (broadly) across many neural circuits? Is it possible to account for this apparent discrepancy between these results. I suspect it comes down to baseline and when it was taken relative to sleep. However, I think it might be nice to make this issue explicit so that other readers do not need to think this through for themselves.

How many of these changes are perhaps due to performance of a sequence as opposed to learning a sequence? Would the human brain be unaffected by making repeated movements without any serial structure?

Minor/Specific comments:

On page 3, the sentence starting "In sum, these….." I did not understand how this related to the earlier paragraph that seemed to discuss learning as opposed to performance.

On page 7, I didn't understand the value provided by this analysis. I am sure it is valuable. I think it could be just better articulated how and what it adds.

Reviewer #3: The manuscript by Griffa et al. investigates neuroplasticity in response to motor learning using novel diffusion MRI methods. The main aim is to overcome previous limitations of conventional diffusion MRI methods in capturing biologically specific brain changes in humans. Longitudinal studies have started to investigated learning-induced neuroplasticity in humans approx. 20 years ago. Diffusion MRI is sensitive to neuroplasticity in grey matter regions and several studies over the past 10 years have consistently shown motor learning-related changes in hippocampal networks using diffusion MRI. The current study leverages the specificity of new MRI hardware (connectome MRI scanner) and software (SANDI) to cell-specific grey matter changes (e.g., astrocytes, neurons). SANDI is one of many new methods proposing increased biological specificity of parameters derived from in vivo diffusion MRI data. Griffa et al. obtained three diffusion MRI scans per participant: before, 30 min. as well as 24h after the first practice session of a new finger sequence task. Results show short-term and transient increases in a cell-specific SANDI marker in hippocampus and motor cortex as well as short-term but persistent (remained at least until 24h) neurite-specific changes in posterior parietal cortex and precuneus. The authors conclude that the results differentiate between plastic (persistent) and non-plastic (transient) changes and provide a mechanistic window into human neuroplasticity relevant to bridge the gap between human and animal neuroscience research. The evidence is based on (1) a well-established learning paradigm (finger sequence learning) suitable for application within the MR-environment, (2) a beneficial combination of diffusion-based MRI and task-based fMRI for localizing relevant brain areas and (3) a remarkable consistency of previous findings (partly from the same group) on hippocampal involvement in early motor sequence learning using conventional DTI methods. Overall, the results appear in line with previous DTI findings and the new methods (SANDI, connectome MR scanner) enabled the discovery of dissociable temporal dynamics in presumably cell- and neurite-specific brain changes underlying the DTI signal - which is indeed a leap forward for the human imaging field and a stimulus for new hypotheses testable with cross-species animal and human MRI studies.

However, there are major concerns related to the validity of conclusions which I describe below:

1. The authors did not include a control group that would allow them to relate SANDI changes to learning (rather than unspecific changes over time). This is unusual, in part, because the sequence learning paradigm has been used for decades using well-controlled study designs. The authors mentioned the consistency of DTI- and fMRI findings with their previous work (that included a non-learning control group), but due to the lack of additional correlations between SANDI changes and learning parameters, the nature of cell- and neurite-specific changes remain elusive. The lack of behavioral correlations is unexpected given the wealth of learning parameters captured by the study design (micro-offline gains, micro-online gains, retention performance…). A proper control group or correlations with learning parameters would be essential to associate brain changes to behavior.

2. With respect to 1., I think that the evidence for concluding "distinct plastic and non-plastic changes" underlying the SANDI signals is low due to the following reasons: first, there are lots of relevant microstructural changes induced during initial skill learning and MRI may only capture the tip of the iceberg. Theory suggests non-linear brain changes during motor skill acquisition (see Hille et al., 2024). Labelling a transient change (fig. 3A) as "non-plastic" ignores the large number of ongoing changes causally relevant for learning (e.g., the clustered addition and pruning of new synaptic spines) but potentially not detectable with diffusion MRI. Second, dissociable time courses should be inferred from direct statistical comparisons of SANDI changes (e.g., using analyses of variance) rather than separate region-wise analyses (as done throughout the manuscript).

3. The authors interpret the increase in soma fraction as potential correlate of activity-induced cell swelling. In the abstract, they note that "We found that learning a motor skill triggered two distinct temporal responses: a transient expansion of cell bodies across all brain regions engaged by the task…". Right-handed participants practiced the finger sequence using their non-dominant left hand and fMRI results indicate task-induced activity in the contralateral right M1 (fig. 2B). However, FSOMA changes appeared in left but not right M1 (figs. 2, 3) which argues against brain activity as driver of FSOMA changes. In addition to that, the hippocampus was more active in the resting periods between practice blocks compared to the actual task execution (fig. 2). It is not clear, however, if this is the results of a deactivation during practice blocks or an activation during rest. Together, the relation between brain activity and cell-specific transient change remains unclear.

4. SANDI offers readouts of cell diameter in addition to the fractions of soma, neurite and free water. Cell diameter was not analysed in the current study but this parameter would be more specific to the proposed cell swelling mechanism (rather than soma fraction).

5. The empirical basis of cell-specific (rather than neurite-specific) plasticity through short-term learning remains to be shown in the intro. Cited studies largely reflect neurite-specific mechanisms but then, why was SANDI and not another diffusion model used in the context of this study?

6. Please also describe the evidence from histological validation studies indicating cell-specific mechanisms as a correlate of SANDI changes.

# Author comment: Author response to Decision Letter 2

**DOI:** 10.1371/journal.pbio.3003861.r003

28 Apr 2026

Attachment Submitted filename: Authors_response.pdf

# Editor report: Decision Letter 2

**DOI:** 10.1371/journal.pbio.3003861.r004

**Authors:** Christian Schnell, PhD, Christian Schnell, PhD, Christian Schnell, PhD

1 May 2026

Dear Valeria,,

Thank you for your patience while we considered your revised manuscript "A mechanistic window onto human neuroplasticity through cell-specific diffusion MRI" for publication as a Short Reports at PLOS Biology. Your revised study has been evaluated by the PLOS Biology editors and the Academic Editor, but not yet by the original reviewers.

Before we will send the manuscript back to the reviewers, however, we would like you to address the editorial comments from the previous decision letter more clearly, because we feel that these haven't been directly addressed. This includes issues about advance. For example, is it actually possible for synaptic changes to be observed after only 30 minutes? Is there any evidence in the literature for this, for example from animal studies? I have pasted the excerpt from the previous decision letter below. I encourage you to add your response to these editorial comments to the rebuttal letter after your response to Reviewer 3's comments, so the reviewers have access to this discussion too.

Given the extent of revision needed, we cannot make a decision about publication until we have seen the revised manuscript and your response to the reviewers' comments. Your revised manuscript is likely to be sent for further evaluation by all or a subset of the reviewers.

In addition to these revisions, you will need to complete some formatting changes, which you will receive in a follow up email. A member of our team will be in touch with a set of requests shortly.

We expect to receive your revised manuscript within 3 months. Please email us (plosbiology@plos.org) if you have any questions or concerns, or would like to request an extension.

At this stage, your manuscript remains formally under active consideration at our journal; please notify us by email if you do not intend to submit a revision so that we may withdraw it.

**IMPORTANT - SUBMITTING YOUR REVISION**

Your revisions should address the specific points made by each reviewer. Please submit the following files along with your revised manuscript:

1. A 'Response to Reviewers' file - this should detail your responses to the editorial requests, present a point-by-point response to all of the reviewers' comments, and indicate the changes made to the manuscript.

*NOTE: In your point-by-point response to the reviewers, please provide the full context of each review. Do not selectively quote paragraphs or sentences to reply to. The entire set of reviewer comments should be present in full and each specific point should be responded to individually, point by point.

You should also cite any additional relevant literature that has been published since the original submission and mention any additional citations in your response.

2. In addition to a clean copy of the manuscript, please also upload a 'track-changes' version of your manuscript that specifies the edits made. This should be uploaded as a "Revised Article with Changes Highlighted" file type.

*Re-submission Checklist*

When you are ready to resubmit your revised manuscript, please refer to this re-submission checklist: https://plos.io/Biology_Checklist

To submit a revised version of your manuscript, please go to https://www.editorialmanager.com/pbiology/ and log in as an Author. Click the link labelled 'Submissions Needing Revision' where you will find your submission record.

Please make sure to read the following important policies and guidelines while preparing your revision:

*Published Peer Review*

Please note while forming your response, if your article is accepted, you may have the opportunity to make the peer review history publicly available. The record will include editor decision letters (with reviews) and your responses to reviewer comments. If eligible, we will contact you to opt in or out. Please see here for more details:

https://blogs.plos.org/plos/2019/05/plos-journals-now-open-for-published-peer-review/

*PLOS Data Policy*

Please note that as a condition of publication PLOS' data policy (http://journals.plos.org/plosbiology/s/data-availability) requires that you make available all data used to draw the conclusions arrived at in your manuscript. If you have not already done so, you must include any data used in your manuscript either in appropriate repositories, within the body of the manuscript, or as supporting information (N.B. this includes any numerical values that were used to generate graphs, histograms etc.). For an example see here: http://www.plosbiology.org/article/info%3Adoi%2F10.1371%2Fjournal.pbio.1001908#s5

*Blot and Gel Data Policy*

We require the original, uncropped and minimally adjusted images supporting all blot and gel results reported in an article's figures or Supporting Information files. We will require these files before a manuscript can be accepted so please prepare them now, if you have not already uploaded them. Please carefully read our guidelines for how to prepare and upload this data: https://journals.plos.org/plosbiology/s/figures#loc-blot-and-gel-reporting-requirements

*Protocols deposition*

To enhance the reproducibility of your results, we recommend that if applicable you deposit your laboratory protocols in protocols.io, where a protocol can be assigned its own identifier (DOI) such that it can be cited independently in the future. Additionally, PLOS ONE offers an option for publishing peer-reviewed Lab Protocol articles, which describe protocols hosted on protocols.io. Read more information on sharing protocols at https://plos.org/protocols?utm_medium=editorial-email&utm_source=authorletters&utm_campaign=protocols

Thank you again for your submission to our journal. We hope that our editorial process has been constructive thus far, and we welcome your feedback at any time. Please don't hesitate to contact us if you have any questions or comments.

Sincerely,

Christian

Christian Schnell, PhD

Senior Editor

PLOS Biology

cschnell@plos.org

===

Excerpt from the previous decision letter:

While we ask you to address all concerns, we specifically we ask you to focus on (i) sharpening the scope and describe the advance of the study in more clarity and (ii) guiding the reader more clearly about what is being shown, how it is learning related, how we can be certain that these are structural changes, and that they relate to some aspect of learning. We think you will be able to address these concerns with textual revisions and (re)analyzing current data, and do not expect you to collect new experimental data.

However, these requests are not simply cosmetic changes. You will see in the reviewer reports that there is some ambiguity in how the reviewers view the advance of you study, so it needs to be very clear what the advance is, and that we can reliably attribute the DTI/SANDI changes to structural change.

# Author comment: Author response to Decision Letter 3

**DOI:** 10.1371/journal.pbio.3003861.r005

3 May 2026

Attachment Submitted filename: Authors_response_auresp_3.pdf

# Peer review: Decision Letter 3

**DOI:** 10.1371/journal.pbio.3003861.r006

**Authors:** Christian Schnell, PhD, Christian Schnell, PhD, Christian Schnell, PhD, Christian Schnell, PhD

20 May 2026

Dear Valeria,

Thank you for your patience while we considered your revised manuscript "A mechanistic window onto human neuroplasticity through cell-specific diffusion MRI" for publication as a Short Reports at PLOS Biology. This revised version of your manuscript has been evaluated by the PLOS Biology editors, the Academic Editor and two of the original reviewers.

Based on the reviews and on our Academic Editor's assessment of your revision, we are likely to accept this manuscript for publication, provided you satisfactorily address the remaining points raised by Reviewer 3. Please also make sure to address the following data and other policy-related requests:

* We would like to suggest a different title to improve its accessibility for our broad audience:

Learning induces transient cell-body expansion and sustained increases in neurite density in the human brain

* Please add the links to the funding agencies in the Financial Disclosure statement in the manuscript details.

* Please include the approval/license number of the ethical approval for the experiments.

* Please include information in the Methods section whether the study has been conducted according to the principles expressed in the Declaration of Helsinki.

* Please provide information whether the participants provided written or verbal consent.

* Please move the methodological details from the supplementary information file to the main manuscript methods sections. We do not have a word count limit.

* DATA POLICY:

You may be aware of the PLOS Data Policy, which requires that all data be made available without restriction: http://journals.plos.org/plosbiology/s/data-availability. For more information, please also see this editorial: http://dx.doi.org/10.1371/journal.pbio.1001797

Note that we do not require all raw data. Rather, we ask that all individual quantitative observations that underlie the data summarized in the figures and results of your paper be made available in one of the following forms:

1) Supplementary files (e.g., excel). Please ensure that all data files are uploaded as 'Supporting Information' and are invariably referred to (in the manuscript, figure legends, and the Description field when uploading your files) using the following format verbatim: S1 Data, S2 Data, etc. Multiple panels of a single or even several figures can be included as multiple sheets in one excel file that is saved using exactly the following convention: S1_Data.xlsx (using an underscore).

2) Deposition in a publicly available repository. Please also provide the accession code or a reviewer link so that we may view your data before publication.

Regardless of the method selected, please ensure that you provide the individual numerical values that underlie the summary data displayed in the following figure panels as they are essential for readers to assess your analysis and to reproduce it: 2A (right panel), 2C (left part of each panel), 3AB, 4 and S1.

NOTE: the numerical data provided should include all replicates AND the way in which the plotted mean and errors were derived (it should not present only the mean/average values).

Please also ensure that figure legends in your manuscript include information on where the underlying data can be found, and ensure your supplemental data file/s has a legend.

Please ensure that your Data Statement in the submission system accurately describes where your data can be found.

* CODE POLICY

Per journal policy, if you have generated any custom code during the course of this investigation, please make it available without restrictions. Please ensure that the code is sufficiently well documented and reusable, and that your Data Statement in the Editorial Manager submission system accurately describes where your code can be found. More information on our Code Policy, what and how to share can be found here: https://journals.plos.org/plosbiology/s/code-availability

Please note that we cannot accept sole deposition of code in GitHub, as this could be changed after publication. However, you can archive this version of your publicly available GitHub code to Zenodo. Once you do this, it will generate a DOI number, which you will need to provide in the Data Accessibility Statement (you are welcome to also provide the GitHub access information). See the process for doing this here: https://docs.github.com/en/repositories/archiving-a-github-repository/referencing-and-citing-content

As you address these items, please take this last chance to review your reference list to ensure that it is complete and correct. If you have cited papers that have been retracted, please include the rationale for doing so in the manuscript text, or remove these references and replace them with relevant current references. Any changes to the reference list should be mentioned in the cover letter that accompanies your revised manuscript.

In addition to these revisions, you may need to complete some formatting changes, which you will receive in a follow up email. A member of our team will be in touch with a set of requests shortly. If you do not receive a separate email within a few days, please assume that checks have been completed, and no additional changes are required.

We expect to receive your revised manuscript within two weeks.

To submit your revision, please go to https://www.editorialmanager.com/pbiology/ and log in as an Author. Click the link labelled 'Submissions Needing Revision' to find your submission record. Your revised submission must include the following:

- a cover letter that should detail your responses to any editorial requests, if applicable, and whether changes have been made to the reference list

- a Response to Reviewers file that provides a detailed response to the reviewers' comments (if applicable, if not applicable please do not delete your existing 'Response to Reviewers' file.)

- a track-changes file indicating any changes that you have made to the manuscript.

NOTE: If Supporting Information files are included with your article, note that these are not copyedited and will be published as they are submitted. Please ensure that these files are legible and of high quality (at least 300 dpi) in an easily accessible file format. For this reason, please be aware that any references listed in an SI file will not be indexed. For more information, see our Supporting Information guidelines:

https://journals.plos.org/plosbiology/s/supporting-information

*Published Peer Review History*

Please note that you may have the opportunity to make the peer review history publicly available. The record will include editor decision letters (with reviews) and your responses to reviewer comments. If eligible, we will contact you to opt in or out. Please see here for more details:

https://plos.org/published-peer-review-history/

*Press*

Should you, your institution's press office or the journal office choose to press release your paper, please ensure you have opted out of Early Article Posting on the submission form. We ask that you notify us as soon as possible if you or your institution is planning to press release the article.

*Protocols deposition*

To enhance the reproducibility of your results, we recommend that if applicable you deposit your laboratory protocols in protocols.io, where a protocol can be assigned its own identifier (DOI) such that it can be cited independently in the future. Additionally, PLOS ONE offers an option for publishing peer-reviewed Lab Protocol articles, which describe protocols hosted on protocols.io. Read more information on sharing protocols at https://plos.org/protocols?utm_medium=editorial-email&utm_source=authorletters&utm_campaign=protocols

Please do not hesitate to contact me should you have any questions.

Sincerely,

Christian

Christian Schnell, PhD

Senior Editor

cschnell@plos.org

PLOS Biology

------------------------------------------------------------------------

Reviewer remarks:

Reviewer #2: The authors have done a great job of revising this work. The third paragraph of the discussion is an example for us all - it very nicely balances the observations with their interpretation. It makes it clear what is known against what is thought.

Reviewer #3: Thank you very much for your responses to my concerns. The manuscript improved significantly. The important behavioral correlations suggest a link between diffusion changes and persistent improvements in performance. In your response to point 3, the lateralization issue was not sufficiently addressed in my opinion. I got the point that left-hand practice engages contralateral AND ipsilateral M1 (consistent with ealier studies), but this does not explain why the highly active right M1 was not affected by diffusion changes. This seems inconsistent with the interpretation that practice-induced increases in brain activity drive neural plasticity. Please provide an explanation for this result.

# Author comment: Author response to Decision Letter 4

**DOI:** 10.1371/journal.pbio.3003861.r007

25 May 2026

Attachment Submitted filename: Authors Response.pdf

# Editor report: Decision Letter 4

**DOI:** 10.1371/journal.pbio.3003861.r008

**Authors:** Christian Schnell, PhD, Christian Schnell, PhD, Christian Schnell, PhD, Christian Schnell, PhD, Christian Schnell, PhD

4 Jun 2026

Dear Dr Della-Maggiore,

Thank you for the submission of your revised Short Reports "Learning engages transient and sustained cellular mechanisms in the human brain" for publication in PLOS Biology. On behalf of my colleagues and the Academic Editor, Edwin Robertson, I am pleased to say that we can in principle accept your manuscript for publication, provided you address any remaining formatting and reporting issues. These will be detailed in an email you should receive within 2-3 business days from our colleagues in the journal operations team; no action is required from you until then. Please note that we will not be able to formally accept your manuscript and schedule it for publication until you have completed any requested changes.

While you attend to those requests to come, please also make sure to reference the source data in the figure legends, for example: "The data underlying this Figure can be found at https://zenodo.org/records/20527091."

Please take a minute to log into Editorial Manager at http://www.editorialmanager.com/pbiology/, click the "Update My Information" link at the top of the page, and update your user information to ensure an efficient production process.

PRESS

We frequently collaborate with press offices. If your institution or institutions have a press office, please notify them about your upcoming paper at this point, to enable them to help maximize its impact. If the press office is planning to promote your findings, we would be grateful if they could coordinate with biologypress@plos.org. If you have previously opted in to the early version process, we ask that you notify us immediately of any press plans so that we may opt out on your behalf.

We also ask that you take this opportunity to read our Embargo Policy regarding the discussion, promotion and media coverage of work that is yet to be published by PLOS. As your manuscript is not yet published, it is bound by the conditions of our Embargo Policy. Please be aware that this policy is in place both to ensure that any press coverage of your article is fully substantiated and to provide a direct link between such coverage and the published work. For full details of our Embargo Policy, please visit http://www.plos.org/about/media-inquiries/embargo-policy/.

Thank you again for choosing PLOS Biology for publication and supporting Open Access publishing. We look forward to publishing your study.

Sincerely,

Christian

Christian Schnell, PhD

Senior Editor

PLOS Biology

cschnell@plos.org
